# Molecular architecture of black widow spider neurotoxins

**DOI:** 10.1038/s41467-021-26562-8

**Published:** 2021-11-29

**Authors:** Minghao Chen, Daniel Blum, Lena Engelhard, Stefan Raunser, Richard Wagner, Christos Gatsogiannis

**Affiliations:** 1 Institute for Medical Physics and Biophysics and Center for Soft Nanoscience, Westfälische Wilhelms Universität Münster, 48149 Münster, Germany; 2 Department of Structural Biochemistry, Max Planck Institute of Molecular Physiology, 44227 Dortmund, Germany; 3 MOLIFE Research Center, Jacobs University Bremen, 28759 Bremen, Germany

**Keywords:** Structural biology, Ion transport, Cryoelectron microscopy

## Abstract

Latrotoxins (LaTXs) are presynaptic pore-forming neurotoxins found in the venom of *Latrodectus* spiders. The venom contains a toxic cocktail of seven LaTXs, with one of them targeting vertebrates (α-latrotoxin (α-LTX)), five specialized on insects (α, β, γ, δ, ε- latroinsectotoxins (LITs), and one on crustaceans (α-latrocrustatoxin (α-LCT)). LaTXs bind to specific receptors on the surface of neuronal cells, inducing the release of neurotransmitters either by directly stimulating exocytosis or by forming Ca^2+^-conductive tetrameric pores in the membrane. Despite extensive studies in the past decades, a high-resolution structure of a LaTX is not yet available and the precise mechanism of LaTX action remains unclear. Here, we report cryoEM structures of the α-LCT monomer and the δ-LIT dimer. The structures reveal that LaTXs are organized in four domains. A C-terminal domain of ankyrin-like repeats shields a central membrane insertion domain of six parallel α-helices. Both domains are flexibly linked via an N-terminal α-helical domain and a small β-sheet domain. A comparison between the structures suggests that oligomerization involves major conformational changes in LaTXs with longer C-terminal domains. Based on our data we propose a cyclic mechanism of oligomerization, taking place prior membrane insertion. Both recombinant α-LCT and δ-LIT form channels in artificial membrane bilayers, that are stabilized by Ca^2+^ ions and allow calcium flux at negative membrane potentials. Our comparative analysis between α-LCT and δ-LIT provides first crucial insights towards understanding the molecular mechanism of the LaTX family.

## Abstract (web summary)

The venom of *Latrodectus* spiders contains seven Latrotoxins (LaTXs), among them α-latrocrustatoxin (LCT) and δ- latroinsectotoxins δ-LIT. LaTXs bind to specific receptors on the surface of neuronal cells and target the molecular exocytosis machinery. Here, the authors present the cryo-EM structure of the α-LCT monomer and the δ-LIT dimer, which reveal that LaTXs are organized in four domains and they discuss the potential oligomerisation mechanism that takes place before LaTXs membrane insertion. Both recombinant α-LCT and δ-LIT form channels in artificial membrane bilayers, that are stabilized by Ca^2+^ ions.

## Introduction

Latrotoxins (LaTXs) are potent high molecular weight neurotoxins from the venom of black widow spiders. The venom contains an arsenal of phylum-specific toxins, including one vertebrate-specific toxin, α-latrotoxin (α-LTX)^1^, five highly specific insecticidal toxins (α-, β-, γ-, δ-, and ε-latroinsectotoxin (LITs))^2, 3^, and one crustacean-specific toxin, α-latrocrustatoxin (α-LCT)^3, 4^. The vertebrate-specific α-LTX causes a clinical syndrome named lactrodectism upon a venomous bite to humans, which is fortunately rarely life-threatening but often characterized by severe muscle cramps and numerous other side effects such as hypertension, sweating, and vomiting^5, 6^.

LaTXs are produced as ~160 kDa inactive precursor polypeptides in venom glands and secreted into the gland lumen. There the final mature 130 kDa toxin is produced by proteolytic processing at two furin sites and cleavage of a N-terminal signal peptide and a C-terminal inhibitory domain^7, 8^. Most of the physiological and molecular biological researches to date have been carried out using the vertebrate-specific toxin α-LTX.

α-LTXs have been shown to form cation-selective pores upon binding to specific receptors on the presynaptic membrane and induce Ca^2+^ influx, thereby mimicking physiological voltage-dependent calcium channels^9, 10^. Ca^2+^ influx activates the exocytosis machinery^11^ and triggers a massive release of neurotransmitters. α-LTX was shown to form also pores on artificial lipid bilayers, which have high conductance for monovalent and divalent cations such as K^+^, Na^+^, Ca^2+^, and Mg^2+^, but are blocked by transition metals and trivalent ions such as Cd^2+^ and La^3+,^^12–16^. Efficient incorporation into biological membranes strictly relies however on the presence of specific receptors^17–19^.

To date, three receptors for α-LTX have been isolated, *i.e*., the cell adhesion protein neurexin (NRX) which binds to the α-LTX in a Ca^2+^-dependent manner^20–22^, the G protein-coupled receptor latrophilin (LPHN or CIRL, stands for Calcium-Independent Receptor of Latrotoxin)^23, 24^ and the receptor-like protein tyrosine phosphatase σ (PTPσ)^25^. With regard to α-LTX, NRX and PTPσ are suggested to provide only a platform for binding and subsequent pore formation events^25–29^. In contrast, Ca^2+^-independent binding to LPHN does not involve oligomerization and channel formation, but direct downstream stimulation of the synaptic fusion machinery^27, 30–32^.

The channel-dependent and independent functions of α-LTX have attracted the attention of neurobiologists for several decades, studying the effects of α-LTX on neurotransmitter release and mechanisms underlying synaptic plasticity. The α-LTX variant LTX^N4C,^^33^, which lacks the ability of pore-formation but retains the full binding affinity to receptors, played a key role in the investigations of α-LTX action. Today, α-LTX is an indispensable tool for stimulating exocytosis of nerve and endocrine cells^29, 34–36^. α-LTXs are furthermore considered to antagonize botulinum poisoning and attenuate the neuromuscular paralysis via synapse remodeling^37^. The surprising structural homology of α-LTX to the glucogen-like peptide-1 (GLP1)-like family of secretagogic hormones might also open opportunities for pharmacological applications in blood glucose normalization and reversal of neuropathies^38^.

Invertebrate LaTXs are less well understood, but considered as promising candidates for the development of novel bio-pesticides. Orthologues of the three receptor classes shown to bind α-LTX are also present in insects^39^. To date, four LaTXs have been cloned, including α-LTX^7^, α-LIT^40^, δ-LIT^41^, and α-LCT^42^. Despite their high specificity, the different LaTXs display a 30–60% sequence identity and are expected to share an overall similar domain organization and membrane insertion mechanism. Low-resolution 3D maps (14–18 Å) of the α-LTX dimer, α-LTX tetramer, and δ-LIT monomer were previously determined using single-particle negative stain and cryoEM^39, 43^, suggesting indeed an overall similar architecture of the different members of the LaTX family.

A structural and mechanistic understanding of LaTX function is a significant priority for the development of novel anti-toxin therapeutics and/or insecticides. However, a high-resolution structure of a LaTX, which is a prerequisite for the understanding of LaTXs’ mechanism of action at molecular detail, has been missing. Here we present a 4.0 Å cryoEM structure of the α-LCT monomer and a 4.6 Å cryoEM structure of the δ-LIT dimer revealing the molecular architecture of LaTX neurotoxins as well as the molecular details of their oligomerization mechanism prior to membrane insertion. In addition we characterized the principal basic pore characteristics of α-LCT, the precursor δ-LIT and δ-LIT channels after reconstitution into planar lipid bilayer.

## Results

### CryoEM structure determination of α-LCT

**Fig. 1:**
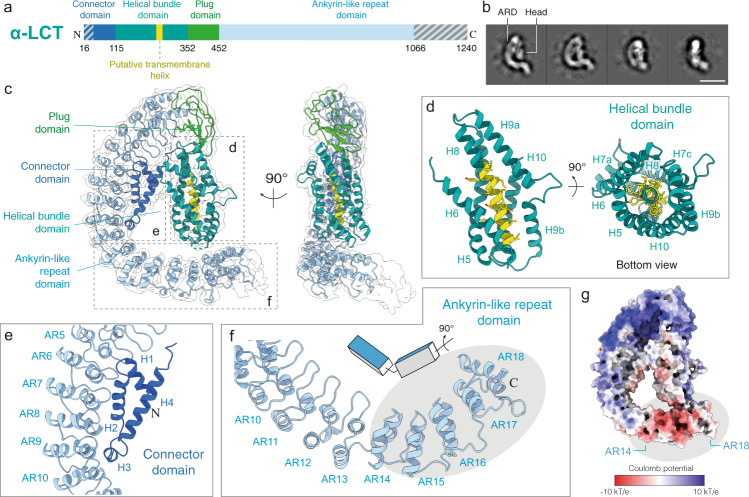
Structure of α-LCT monomer. **a** Domain organization of mature α-LCT. Gray diagonal lines indicate regions not resolved in the cryoEM density. **b** Representative reference-free two-dimensional class averages. Scale bar: 10 nm. **c** Side views of the α-LCT monomer superposed with the EM map (transparent) contoured at 10σ. Domains are depicted in the same colors as in (**a**). **d** Close-up view of the helical bundle domain. The front helix (H7) is not shown in the left image for clarity. **e** Close-up view of the interface between CD (H1-3) and ARD (AR5-10). **f** Close-up view of the ARD C-terminal tail. The gray ellipse indicates the last five ARs (AR14-18). Note the change in orientation **g** Electrostatic potential calculated in APBS. Red: −10 kT/e; Blue: +10 kT/e. PD:plug domain; HBD:helical bundle domain; CD: connector domain; ARD: ankyrin-like repeat domain.

We recombinantly expressed the mature α-LCT (amino acids 16–1240) from *Latrodectus tredecimguttatus* (Mediterranean black widow) (UniprotKB ID: Q9XZC0 (LCTA_LATTR)) (Fig. 1a and Supplementary Fig. 1) in insect cells using the MultiBac system and purified it using a combination of affinity and size exclusion chromatography to obtain a monodisperse sample for cryoEM single particle analysis (Supplementary Fig. 2a, b). The cryoEM sample showed a homogeneous set of characteristic G-shaped flat particles, corresponding to soluble monomers of the 130 kDa mature α-LCT complex (prepore state; before membrane insertion). The G-shaped particle is composed of a C-like curved region, corresponding to the long C-terminal domain of ankyrin-like repeats (ARs), that is engulfing a central compact head region (Fig. 1b, Supplementary Fig. 2c).

Subsequent image processing and 3D classifications revealed an inherent flexibility between both regions. The N-terminal head is orientated perpendicular and in close vicinity to the tail of the AR region in the best-resolved class (compact conformation), but shows a continuous movement and is tilted away the tail of the AR-domain in less well-resolved classes, resulting in less compact conformations (Supplementary Figs. 2d, e, 3a).

The α-LCT monomer in the compact conformation adopts a flat architecture and is 130 Å long and 30 Å wide. The path of the polypeptide is clear in the density map, allowing us to build an atomic model covering 81% of the sequence of the molecule (residues 48–1066, except two disordered loop regions 226–232, 349–360) (Supplementary Fig. 4a). We deleted side-chain atoms beyond Cβ in regions where side-chain density was only rarely evident. The C-terminal end of the ARs domain including the last four ankyrin repeats is not well resolved. The local resolution is highest in the N-terminal head region (Supplementary Fig. 2h).

### Architecture of the soluble α-LCT monomer

The resulting atomic model reveals that the N-terminal head region is composed of three domains: a four-helix domain at the N-terminal end, termed here as connector domain (residues 48–115); a central helical bundle domain (residues 116–352), and a short β-sheet domain, linking the helical bundle domain with the AR domain, termed here as plug domain (residues 353–452) (Fig. 1a, c, Supplementary Fig. 5, Supplementary Movie 1).

The helical bundle domain shows a novel fold of a six-helix bundle, with five parallel aligned helices assembling into a cylindrical structure (H5-7, H9-10), encircling a central α-helix (H8) (Fig. 1d). The conserved helix H8 contains many hydrophobic residues and is predicted to act as transmembrane region of the tetrameric LaTX pore (Supplementary Fig. 6d). The surrounding helices shield the hydrophobic surface of H8 within the cylindrical bundle and protect it from the aqueous environment (Supplementary Fig. 7a, b). The helical bundle domain is expected to undergo severe conformational changes during pore formation to allow exposure of the transmembrane helix H8 and transition of the toxin from a soluble monomer to a transmembrane tetramer. Interestingly, helices H7 and H9 are kinked and interrupted by two (residues 191–196, 203–211) and one (residues 296–304) short loops, respectively. Such short breaks interrupting long α-helices in close vicinity to the putative transmembrane regions, were shown in other α-helical pore forming toxins to provide the necessary flexibility for major conformational changes towards membrane insertion^44^.

The long and curved C-shaped C-terminal AR domain consists of 22 ankyrin-like repeats, accounting for two thirds of the sequence of α-LCT. In total, the first 18 out of 22 ankyrin-like repeats (ARs) were resolved in the present map. Interestingly, there is a redirection of orientation of the ARs at the loop (residues 890–902) connecting AR13 and AR14. ARs14-18 are rotated almost 90 degrees compared to ARs1-13 along the long axis of the domain (Fig. 1f). This arrangement goes along with a characteristic bipolar charge distribution, with ARs1-13 dominantly positively charged and the tail of ARs14-18 displaying a prominent negatively charged patch (Fig. 1g, Supplementary Fig. 6a). The C-terminal tail of the AR-domain is considerably close to the helical bundle domain, but the I226-A232 loop of the helical bundle that connects H7 and H8 and might cross-bridge the 5 Å distance between both domains, is not resolved in the cryoEM density and we were also not able to find strong candidates for electrostatic and hydrophobic interactions. Furthermore, 3D classification (Supplementary Fig. 2d) of the dataset further suggests high flexibility in this region and variable distances between both domains, rendering the possibility of stable large scale interactions between the helical bundle domain and AR-domain rather unlikely (Supplementary Fig. 7c).

The connector domain at the N-terminal end forms the lower interface connecting the central helical bundle with the AR-domain (Fig. 1c, e). Helices H1, H2, H4 and the short helix H3 of the connector domain, assemble into a flat triangle structure, which is attached to the inner curved surface of the AR-domain and interacts with ARs 6-10. Most interaction surface to AR-domain is provided by H2, whereas the short helix H3 is directly positioned in close proximity to the loop connecting AR9 and AR10 and parallel aligned to the helices of AR9 and AR10 (Fig. 1e). The residues involved in this interface are only conserved in the AR-, not in the connector domain (Supplementary Fig. 7d, e). They are mainly hydrophobic, suggesting a major role of hydrophobic interactions. Helix H2 of the connector domain is hydrophobic in our structure and interestingly, this helix has been also predicted as the second transmembrane region of the insecticidal δ-LIT (Supplementary Fig. 6h), but not predicted as such from the sequences of other latrotoxin family members, such as α-LCT (Supplementary Fig. 6d).

The plug-domain covalently links the primary sequence of the helical bundle with the AR-domain, positioning the helical bundle directly below AR1 (upper interface) (Fig. 1c, Supplementary Fig. 7f–h). The plug-domain is organized in two layers: a region of several flexible loops and a core region of four β-strands that is attached to H5 and a short loop between H8 and H9 of the helical bundle (Supplementary Fig. 7i). The plug-domain plays an important role in the oligomerization of the complex prior complex formation, which will be discussed in detail in the next section.

### CryoEM structure of inactive precursor soluble δ-LIT dimer

**Fig. 2:**
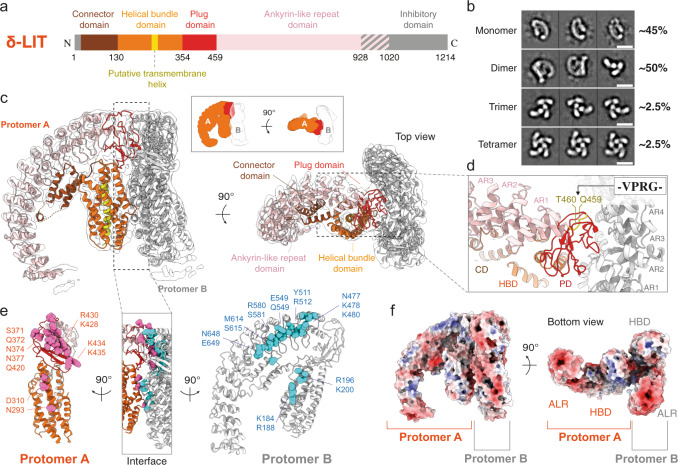
Structure of δ-LIT dimer. **a** Domain organization of full-length precursor δ-LIT. Disordered regions in the cryoEM map are indicated in gray diagonal lines and boxes. **b** Representative two-dimensional class averages of each oligomeric state. Scale bar: 100 Å. **c** Side and top view of the δ-LIT dimer superposed with the EM map (transparent) contoured at 10σ. Domains of protomer A are depicted in the same colors as in (**a**); protomer B is colored in gray. **d** Close-up view of the PD-ARD dimerization interface. The position of the four amino acid insertion variant (VPRG) is indicated and colored in yellow. **e** Side view of the dimerization interface. Protomers A and B are rotated 90° to left and right, for better clarity. Polar and charged candidate residues (<5 Å to the opposite protomer) are shown as spheres and colored in cyan and pink. **f** Electrostatic potential calculated in APBS. Red: −10 kT/e; Blue: +10 kT/e. PD:plug doman; HBD:helical bundle domain; CD: connector domain; ARD: ankyrin-like repeat domain.

In subsequent experiments, we were not able to induce oligomerization of α-LCT and trigger insertion into liposomes for further visualization of pore formation events as previously described for α-LTX^43, 45^. To provide further insights into the LaTX family, we then focused on the insecticidal δ-LIT (amino acids 1-1214 from *Latrodectus tredecimguttatus*, UniprotKB ID: Q25338 (LITD_LATTR)). As expected, mature δ-LIT was toxic for our insect cell cultures and therefore, we expressed, purified, and subjected to cryoEM analysis the precursor uncleaved inactive toxin (Supplementary Fig. 8a–c). The precursor toxin contains an additional N-terminal signal peptide (residues 1–28) and a C-terminal inhibitory domain (α-LCT residues 1037–1214), compared to the matured form (Fig. 2a). Albeit we performed the cryoEM analysis with the same procedure used for mature α-LCT, subsequent processing did not only reveal G-shaped monomers, as was the case for mature α-LCT, but also higher order oligomers. In particular, reference-free 2D classifications revealed approximately 45% monomers, 50% dimers, 2.5% trimers and 2.5% tetramers (Fig. 2b, Supplementary Fig. 8e–h). Interestingly, such particle populations of oligomers were not observed in negative-stain EM (Supplementary Fig. 8c), indicating that the interactions are rather weak and dilution of the sample which is necessary for negative-stain EM, as well as the low pH of the stain might induce dissociation of the oligomers. We were finally able to obtain a map of the δ-LIT dimer at 4.6 Å average resolution from 81,192 particles, with no symmetry imposed (Supplementary Fig. 9).

Based on the structure of α-LCT monomer, we were able to build a molecular model of the δ-LIT dimer, including residues 50–928 for both protomers (Fig. 2c, Supplementary Fig. 4b, Supplementary Movie 2). As expected, the structure of the protomer of δ-LIT is very similar to that of α-LCT (sequence identity 39%), showing the characteristic G-shaped architecture and domain organization. δ-LIT displays however a shorter repetitive C-terminal AR-domain, containing only 15 ARs instead of 22 in α-LCT.

The path of the polypeptide is clear for all four domains, i.e., connector domain, helical bundle domain, plug domain, and AR-domain, except for a few loop regions (residues 91–92, 99–105, 237–245, 355–365) in protomer A and (237–244, 356–361) in protomer B, the last (15th) AR and the complete signal peptide and inhibitory domain in both protomers (Fig. 2c). These structural regions were not resolved in the cryoEM density map, probably because they are disordered or highly flexible. Due to limited resolution, we deleted most of the side-chain atoms beyond Cβ in the molecular model unless the electron density was sufficiently clear with regard to bulky side chains.

The δ-LIT dimer is formed with the two protomers rotated 90 degrees relative to each other and the plug domain of protomer A plugging from the side into a cleft formed by ARs 1-6 of protomer B (Fig. 2c). The protomers A and B are in basically the same conformation, but the small differences, mainly due to the flexibility of the helical bundle domain, precluded successful C2 symmetrization of the particle (Supplementary Fig. 3e).

The plug-domain has a hemispherical architecture matching the cleft formed by the 1st–6th ARs of the AR-domain (Fig. 2d), suggesting an induced fit and shape complementarity as the basis for the interaction. We found clusters of 17 polar and charged residues on the plug-domain of protomer A and 18 polar and charged residues on the cleft of protomer B, that may be involved in this interface (Fig. 2e, Supplementary Table 1). In previous studies, the α-LTX variant LTX^N4C^ with an insertion of a thrombin site (VPRG) at the linker peptide connecting the plug domain with the AR-domain was first introduced to functionally characterize the N- and C-terminals individually^33^. Although cleavage was not successful, this LaTX variant was shown later to retain its binding affinity to receptors but lose its ability to oligomerize into tetramers and form pores^27^. This variant played a key role in understanding the dual mode of action of LaTXs. The corresponding position of this insertion is highlighted on the molecular model of δ-LIT (Fig. 2d). This insertion is not positioned directly at the dimerization interface, but in close proximity to the loops of the plug-domain involved in the interaction and might thus disturb the overall shape complementarity and/or induce a shift of positions and a mismatch between the residues involved in this interface, thereby blocking dimerization.

Besides the main interaction between the plug-domain of protomer A and ARs 1–6 of protomer B, there is a less pronounced interaction between the helical bundle domains of both protomers, involving H9 of protomer A and H6/H7a of protomer B (Fig. 2e, Supplementary Table 1). Whereas H9 is interrupted in protomer A and in the structure of α-LCT by a short loop in its middle, in protomer B (thus most probably upon dimer formation), this loop folds helically to straighten and complete H9 (Supplementary Fig. 3f, Supplementary Fig. 4b).

Interestingly, because δ-LIT is lacking seven terminal ARs, the surface of the AR-domain does not display a clear bipolar charge distribution as in α-LCT (Fig. 2f, Supplementary Fig. 6e). However, in both LaTXs, the terminal tail of the AR-domain is clearly negatively charged (Supplementary Fig. 6a, e).

We further processed the subset including δ-LIT monomers, and upon 3D classification, we finally obtained two 3D reconstructions at a nominal resolution of 8.8 Å and 12 Å, respectively (Supplementary Fig. 10a). The molecular model of δ-LIT obtained from the dimer was then rigid body fitted in the 3D volumes (Supplementary Fig. 10b, c). This comparison revealed an additional globular density at the C-terminal tail of both reconstructions that is not occupied by the model, which can be interpreted as the C-terminal 22 kDa inhibitory domain. The two δ-LIT monomer reconstructions show very similar conformations with the protomers of the dimer, with small differences in the orientation of the helical bundle- and AR-domain, suggesting inherent flexibility of the particle (Supplementary Fig. 10d). We were not however able to obtain 3D reconstructions of the δ-LIT trimers and tetramers due to the preferred orientation of the top views towards the air–water interface (Supplementary Fig. 8g, h). Nevertheless, according to our knowledge, this is the first observation of a LaTX trimer as an intermediate state, towards the formation of the tetramer.

### Structural differences between δ-LIT and α-LCT

**Fig. 3:**
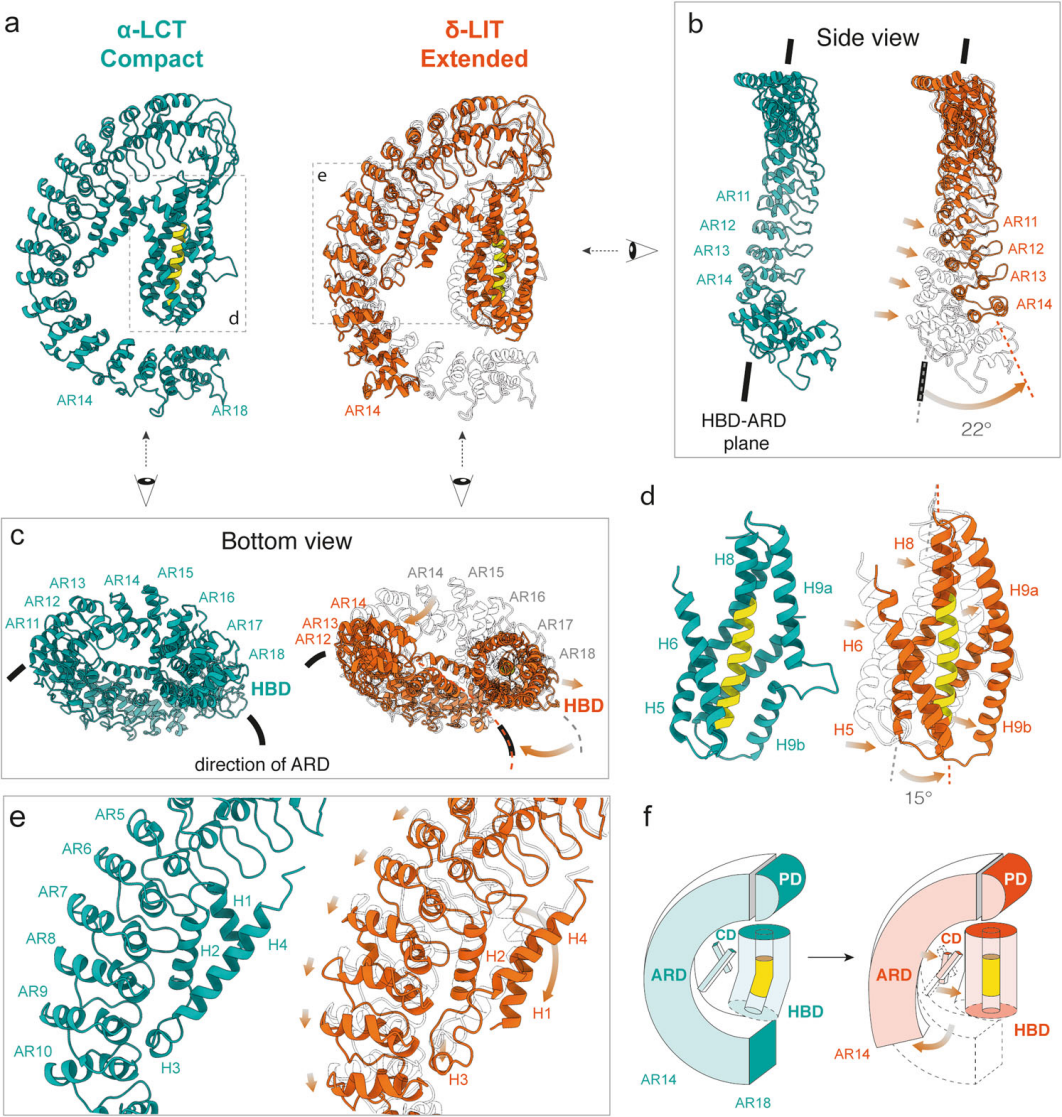
Conformational changes during dimerization. **a** Side-by-side comparison of α-LCT (compact state, sea green) and δ-LIT (extended state, extracted from the dimer, orange) superposed with α-LCT (transparent). **b** Front view of the ARD. Arrows indicate domain motion during dimerization. **c** Bottom views of the compact and extended states. The bottom part of the cylindrical (HBD) is exposed in the extended state. **d** Magnified view of the HBDs. The front helix (H7) is not shown for clarity. The H9a-H9b loop folds helically to complete H9 in the extended state. **e** Magnified view of the interface between the connector- and the AR-domain. **f** The schematic diagram illustrates the conformational change. PD:plug domain; HBD:helical bundle domain; CD: connector domain; ARD: ankyrin-like repeat domain.

In comparison to the compact conformation of the truncated α-LCT monomer, full-length precursor δ-LIT (both in the monomer and dimer state) shows a different, rather extended conformation and a larger distance between the helical bundle- and the AR-domain. Here, we used the model obtained by the best resolved δ-LIT density (protomer A in the δ-LIT dimer, (4.6 Å)), for direct comparison with the α-LCT. In particular, the outermost helix H9 and the overall helical bundle domain are straightened, the helical bundle of each protomer is further tilted 15 degrees away from the long axis of the AR-domain and the distance between the helical bundle domain and the shorter AR-domain is substantially longer. Notably, the AR-domain of δ-LIT is significantly less curved (Fig. 3a) and its C-terminal tail is further twisted and positioned outside the helical bundle domain and ankyrin-like repeat domain (HBD-ARD) plane (Fig. 3b). As a consequence, the bottom part of the helical bundle domain becomes exposed in this case (Fig. 3c).

The enlarged distance between AR-domain and helical bundle domain, also requires a more extended conformation of the connector domain, which is further stretched in δ-LIT, but still bridges both domains. This results into repositioning of H1 and unfolding of the lower half of H4 of the connector domain (Fig. 3e). These differences between the compact α-LCT and the extended δ-LIT are summarized in Supplementary Movie 3 and a schematic diagram in Fig. 3f. Interestingly, the less well-resolved 3D class of α-LCT can be considered as flexible intermediate between both structures (Supplementary Fig. 3g, h, Supplementary Movie 3) and the two low-resolution 3D reconstructions of the δ-LIT monomer further suggest inherent flexibility between the helical bundle domain and the AR-domain.

In general, α-LCT appears more compact, due to the seven additional terminal ARs, possibly allowing additional interactions between the AR-domain and the flexible central helical bundle. This might explain the different oligomerization properties observed for both molecules under identical cryoEM conditions. The directional change observed in the AR-domain of the extended δ-LIT protomer, would also result in the exposure of the helical bundle domains, even for LaTXs with significantly longer AR-domains such as α-LCT or α-LTX (Fig. 3c, right panel).

It should be noticed that the previous low-resolution 3D reconstructions on α-LTX and δ-LIT show an overall different LaTX architecture. The previous 2D cryoEM class averages of α-LTX^43^ and the present 2D classes of δ-LIT dimers and tetramers (Fig. 2b) display nevertheless clear similarities, indicating structure conservation. Taking into account in addition the high sequence similarity within the LaTX family, we rather conclude that the earlier LaTX reconstructions determined more than two decades ago, are apparently to some extent affected by the previous bottlenecks of the technique and the significantly lower signal to noise ratio in the cryoEM micrographs. The members of the LaTX family share an overall common domain organization and architecture.

### Electrophysiological characteristics of the pore-forming precursor δ-LIT in comparison with the mature δ-LIT and α-LCT

We further performed electrophysiology studies to demonstrate pore-formation activity of the recombinant proteins and provide a detailed characterization for the less well-studied invertebrate LaTX channels. One important aspect herein is the role of the C-terminal domain in channel formation, since its cleavage is required for activation of the toxins. Therefore, we additionally prepared mature truncated δ-LIT for further functional comparisons with precursor full-length δ-LIT and mature truncated α-LCT. The inherent cleavage sites were however not recognized by furin protease and therefore we inserted two additional cleavage sites into the sequence of precursor δ-LIT, before the N-terminal- (residue 29) and after the AR-domain (residue 1019) followed by proteolysis treatment after expression (Supplementary Fig. 11). The two mature toxins and precursor δ-LIT were then reconstituted into planar lipid bilayer and voltage-dependent (${V}_{{{{{{{\rm{mem}}}}}}}}$, membrane potential) membrane currents were recorded at single-channel resolution as previously described^46^.

**Fig. 4:**
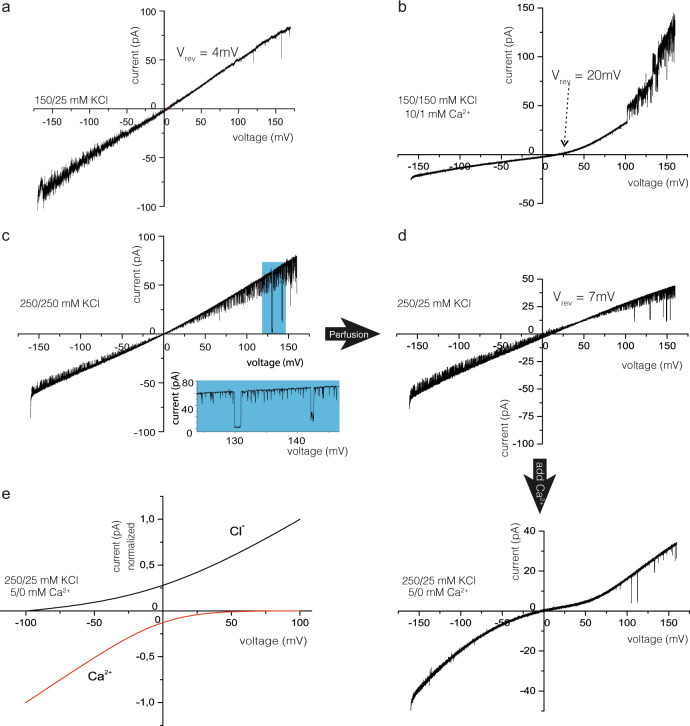
Calcium induced ion-channel properties of reconstituted δ-LIT variants within lipid bilayers. Current–Voltage ramp recordings at different *cis/trans* buffer conditions. **a** Precursor δ-LIT shows in asymmetric KCl buffer a noisy linear current–voltage relation with reversal potential of *V*_rev_ = +4 mV, indicating a slight cation selective channel (${P}_{{{{{{{\rm{K}}}}}}}^{+}}/{P}_{{{{{{{{\rm{Cl}}}}}}}}^{-}}$=1.25). A total of *n*=6 recordings similar to (**a**) from 2 different precursor δ-LIT preparations have been conducted. **b** With symmetric KCl and added asymmetric calcium the precursor δ-LIT channel displays a *V*_rev _= +20 mV, characteristic now for a highly Ca^2+^ selective channel (${P}_{{{{{{{{\rm{Ca}}}}}}}}^{2+}}/{P}_{{{{{{{{\rm{Cl}}}}}}}}^{-}}=18$), demonstrating a dramatic property change induced by the presence of Ca^2+^ ions. A total of *n* = 6 recordings similar to (**b**) from two different precursor δ-LIT preparations have been conducted. **c** The current–voltage relation of the mature δ-LIT is again linear but with low current noise and frequent, defined gating from the open towards the closed state. (displayed in extension box). **d** Next *trans* chamber perfusion within the same experiment. The *V*_rev_ = 7 mV value discloses cation selective properties $({P}_{{{{{{{\rm{K}}}}}}}^{+}}/{P}_{{{{{{{{\rm{Cl}}}}}}}}^{-}} = 1.47)$ preserved within the mature variant. A total of *n* = 6 recordings similar to (**c**, **d**) from two different δ-LIT preparations have been conducted. With asymmetric KCl and *cis* added calcium (lower panel, no calcium addition *trans*) the precursor δ-LIT channel displays an asymptotic sine current–voltage relation with a zero-current crossing at *V*_rev_ = 0 mV. Extrapolation of the tangent to zero net current yields ${V}_{{{{{{{\rm{rev}}}}}}}}=\approx 40{{{{{{\rm{mV}}}}}}}$ and a very high calcium selectivity of the mature δ-LIT channel (${P}_{{{{{{{{\rm{Ca}}}}}}}}^{2+}}/{P}_{{{{{{{\rm{K}}}}}}}^{+}}{/P}_{{{{{{{{\rm{Cl}}}}}}}}^{-}}\cong 600/1.47/1$). A total of n ≥ 6 recordings similar to (**d**; lower panel) from 2 different δ-LIT preparations have been conducted. **e** Calculated GHK current–voltage relation using the experimental relative permeabilities from (**d**; lower panel) and the experimental concentration of ${{{{{{{\rm{Ca}}}}}}}}^{2+}$,${{{{{{\rm{K}}}}}}}^{+}$ and ${{{{{{{\rm{Cl}}}}}}}}^{-}$ions in the *cis* and *trans* compartment (see Supplemental Fig. 12 for details).

Precursor δ-LIT spontaneously inserted into the lipid bilayer and formed open channels as obvious from the observed large voltage induced currents (Supplementary Fig. 12a, c). With asymmetric (*cis/trans*) 150/25 mM KCl buffer conditions, the reversal potential (*V*_rev_) was *V*_rev_=4 ± 1 mV (SD of the linear *i*/*v*-curve fit) yielding ${P}_{{{{{{{\rm{K}}}}}}}^{+}}/{P}_{{{{{{{{\rm{Cl}}}}}}}}^{-}}$=1.25^47, 48^, demonstrating only marginal selectivity to ${{{{{{\rm{K}}}}}}}^{+}$over ${{{{{{{\rm{Cl}}}}}}}}^{-}\,$ions (Fig. 4a). However, upon addition of 10/1 mM CaCl_2_ (*cis/trans*)) gradient in symmetrical 150/150 mM KCl buffer, we observed an approximate five-fold increase in the reversal potential (${V}_{{{{{{{\rm{rev}}}}}}}}=20\pm {2,5}{{{{{{\rm{mV}}}}}}}$ (SD of the linear *i/v*-curve fit) (Fig. 4b)) revealing that the precursor δ-LIT voltage-activated channel preferentially conducts ${{{{{{{\rm{Ca}}}}}}}}^{2+}$ ions (${P}_{{{{{{{{\rm{Ca}}}}}}}}^{2+}}/{P}_{{{{{{{{\rm{Cl}}}}}}}}^{-}}$=18) and the current–voltage relation became rectifying. Moreover, calcium reduced the root-mean-square (rms) values of the current noise drastically. The high rms current noise values in the recordings with the precursor δ-LIT channel are due to a seemingly additional current noise presumably arising from a less stable channel conformation with very fast (<10 ns) and probably incomplete brief open/closing transitions of the channel. This could not be resolved in time and amplitude by our technique and thus appeared as a broad current band with significantly larger rms values than arising from the instrumental set-up only. The mean main conductance as determined from all point current histograms at different voltages was ${\bar{G}}_{{{{{{{\rm{main}}}}}}}}=330\pm 36{{{{{{\rm{pS}}}}}}}\,({{{{{{\rm{SD}}}}}}},{N}=12)$

The current–voltage relations of membrane inserted mature δ-LIT in symmetrical 250/250 mM KCl buffer showed a linear course with high gating activity (Fig. 4c). In contrast to the precursor variant, we observed low noise currents with structured gating which could be resolved in amplitude and time. Similarly to the precursor-, mature δ-LIT channels display also only a slight cation selectivity (${P}_{{{{{{{\rm{K}}}}}}}^{+}}/{P}_{{{{{{{{\rm{Cl}}}}}}}}^{-}}$=1.47, Fig. 4d) after establishing the 250/25 mM KCl gradient. Addition of 5 mM Ca^2+^ ions to the *cis* side of the bilayer changed however the properties of the mature δ-LIT channel completely. The asymptotic-sine course of the current–voltage relation increased at both negative and positive command voltages (*V*_cmd_) while crossing the zero voltage with zero net current (Fig. 4d upper panel). To further analyze this, we applied the widely used Goldman–Hodgkin–Katz (GHK) approach^47–49^ (see Supplemental for details). It turned out that the course of the current–voltage relation in the lower panel of Fig. 4d can be explained if the currents at negative *V*_cmd_ are carried from *cis* to *trans* mainly by Ca^2+^ ions and at positive *V*_cmd_ predominantly by Cl^−^ ions (Fig. 4e; see Supplemental for details). The analysis of current traces revealed two open channel states with mean amplitudes of ${\bar{G}}_{1}=25\pm 2.8{{{{{{\rm{pS}}}}}}}$ and ${\bar{G}}_{2}=180\pm 24.8{{{{{{\rm{pS}}}}}}}$ (SD of the histogram fit) corresponding to a pore restriction diameter of about 0.8 nm^48, 50^ (Supplementary Fig. 12e, f) (*N*=15) significantly smaller than the one calculated for the precursor δ-LIT channel (~1.5 nm see Supplementary Fig. 12b). Thus the mature δ-LIT channel appears to form a denser, stabilized conformation compared to its precursor variant. Additionally, the mature δ-LIT seems to act like a complete rectifier, which in the presence of Ca^2+^, depending on membrane polarization, allows mainly either flux of calcium −*V*_mem_ or chloride currents (+*V*_mem_) from *cis* to *trans*. In this context, it seems important that both the precursor δ-LIT and the mature δ-LIT are incorporated into the bilayer predominantly in a unidirectional manner, with a regulative Ca^2+^-binding site on the *cis* side. Strong rectifying current–voltage relations were observed in all bilayer experiments in the presence of Ca^2+^ ions added on the *cis* side (Fig. 4b, d lower panel).

Surprisingly, mature α-LCT incorporates into the bilayer membrane and forms channels only in the presence of Ca^2+^ ions (*cis*) (Supplementary Fig. 12g). The analysis of the single-channel traces (Supplementary Fig. 12h, i) revealed, similar to mature δ-LIT, two distinct open channel states ${\bar{G}}_{1}=78\pm 5.6{{{{{{\rm{pS}}}}}}}$ and ${\bar{G}}_{2}=180\pm 17.3{{{{{{\rm{pS}}}}}}}$ (*N*=7) (SD, from the fit of amplitude histogram, Supplementary Fig. 12i). Besides the 10/1 mM CaCl_2_ gradient, the α-LCT channel does not show, in contrast to δ-LIT, any preference for the involved anions and cations (${V}_{{{{{{{\rm{rev}}}}}}}}=0\pm 2.1{{{{{{\rm{mV}}}}}}}$ (Supplementary Fig. 12g). In comparison to mature δ-LIT (Fig. 4f), the α-LCT channel displays a higher current noise level in the recordings and surprisingly, considering the slightly asymptotic sine course of the current–voltage relation (Supplementary Fig. 12g), the α-LCT apparently also harbors rectifying properties, making it likely that rectified currents of Ca^2+^ ion and Cl^−^ ions may convey similar to mature δ-LIT.

To sum up, Ca^2+^ ions appear to further stabilize precursor and mature LaTX oligomers after incorporation into the membrane resulting in a rectifying calcium selective channel allowing calcium flux at negative membrane potentials. High Ca^2+^ permeability was previously also shown for native δ-LIT channels in locust muscle membrane and artificial bilayer, but not for truncated recombinant variants^41^. Provided that as a result of the predominantly unidirectional insertion the *cis* side corresponds to the extracellular side of the respective channel, δ-LIT and α-LCT display ion-channel characteristics similar to Ca^2+^-release channels^51^.

### Formation of an insertion-competent prepore complex

The reference free 2D class averages of monomers, dimers, trimers and tetramers identified in the δ-LIT cryoEM dataset (Fig. 2b) suggest a general sequential oligomerization mechanism of LaTXs towards the formation of a soluble tetrameric complex prior membrane insertion. The bent cleft at the upper part of the AR-domain of a protomer is employed as a binding site for the hemispherical plug domain of an adjacent protomer, with both molecules rotated 90 degrees relative to each other. Four LaTX molecules dock into each other in a sequential circular ring-fashion via 1/2/3-mers to eventually form tetramers (Supplementary Fig. 13a), suggesting that the tetramer is not exclusively formed upon assembly of two dimers, as previously proposed^45^.

**Fig. 5:**
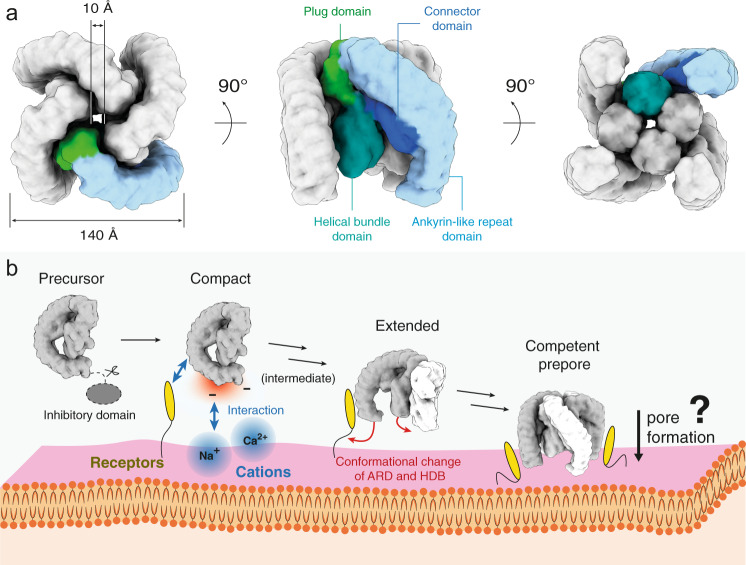
Latrotoxin tetramer assembly prior membrane insertion. **a** Simulated volume of a soluble δ-LIT insertion-competent tetramer. **b** Proposed model of latrotoxin action at the presynaptic membrane prior membrane insertion. The G-shaped toxin is activated upon cleavage of the 200aa C-terminal inhibitory domain. The present study reveals a characteristic bipolar charge distribution with the tail of the complex displaying a prominent negatively charged patch. We therefore propose that the toxin is orientated with its tail facing the positively charged outer surface of the presynaptic membrane (alternative scenarios canot be ruled out; see Supplementary Fig. 16). It is likely that toxin-receptor interactions trigger the oligomerization of the toxin and assembly of a competent prepore tetramer. The dimer is thereby first formed with the two protomers rotated 90 degrees relative to each other. The receptor-binding site is probably positioned at the C-terminal tail of the toxin (see “Discussion”). Structures of receptor-toxin complexes are however still lacking and further studies are now required to shed light into these interactions. Latrotoxins with longer C-terminal tails are expected to undergo large conformational changes during assembly of the prepore tetramer (see Discussion). Subsequently, a calcium selective pore is inserted into the presynaptic membrane, the underlying mechanism is however still unclear.

Based on our data, we generated a 3D model of the tetramer by arranging the δ-LIT dimer (“extended state”) according the 2D class averages of the top view of the tetramer. The resulting 3D model of the tetramer is approximately 140 × 140 Å large and has a height of approximately 120 Å, with an overall striking configuration resembling a four-finger crane claw, with each curved AR-domain resembling a finger of the crane claw (Fig. 5a).

In the resulting model, the four cylindrical helical bundle domains are surrounding a central 10 Å diameter channel, which agrees excellent with the 2D class averages of the soluble δ-LIT tetramer (Fig. 2b) and the previous 2D class averages of the soluble α-LTX tetramer^43^. The putative transmembrane helices are still however completely shielded from the aqueous environment within the respective helical bundle domains. Nevertheless, the bottom part of the helical bundle domains is exposed in this arrangement. We therefore propose that the tetramer, shown here, composed of extended protomers, resembles an insertion-competent prepore state of the toxin.

Assembling the tetramer in a similar manner from α-LCT compact protomers with 22 ARs results instead into a completely closed crane claw (Supplementary Fig. 13b). In this scenario, the AR-domains shield the central helical bundle domains from both sides and the central channel is closed. The resulting compact structure does not match however the 2D class averages of LaTX tetramers (Fig. 2b and Orlova et al.^43^) and there are severe clashes between the AR-domains of adjacent subunits. Note that in previous 2D class averages of α-LTX tetramers with 22 ARs^43^, the central helical bundle domains are also exposed, suggesting that the single protomers are in the extended conformation. This suggests that α-LCT has to undergo a conformational switch from the compact to extended conformation during the oligomerization process and not after formation of the tetramer. Low-resolution 3D classes of the α-LCT monomer resembling intermediate states between the compact and the extended conformation further support this scenario (Supplementary Fig. 3g, h, Supplementary Movie 3). Interestingly, in contrast to α-LCT, the δ-LIT monomer with the shorter C-terminal tail of 15 instead of 22 ARs is already in the extended state. A conformational change (compact to extended) is apparently only required for LaTXs with longer C-terminal domains. α-LCT shows an intriguing bipolar charge contribution, which is present also in α-LTX which has also 22 ARs, but however less pronounced in δ-LIT with 15 ARs (Supplementary Fig. 14, Supplementary Fig. 6a, e). The claw tips (lower ends of the AR-domains) are however clearly negatively charged in all LaTXs.

## Discussion

### Oligomerization characteristics and function of the inhibitory domain

Albeit extensive efforts, also in presence of artificial membranes, we were not able to determine in vitro, in absence of receptors, factors controlling the oligomerization and subsequent pore formation process efficiently for further visualization of the pore formation events. Interestingly, we observed the various populations of prepore oligomers of precursor δ-LIT (2.5% of the particles) only in cryoEM samples (Supplementary Fig. 8h) but not during negative stain EM analysis (Supplementary Fig. 8c). The oligomerization occurred in buffer containing EDTA, and the substitution of EDTA with Mg^2+^ or Ca^2+^ did not affect the sample (Supplementary Fig. 15). This observation corresponds to a previously reported study^45^, suggesting oligomerization of δ-LIT as a process independent of divalent cations. Besides cations, we tried in addition to induce oligomerization/pore-formation of both α-LCT and δ-LIT in presence of detergents or various lipid compositions^52^ and even simulate interactions with the air–water interface by bubbling or pouring over a glass rod^53^. These factors did not have a clear effect on toxin oligomerization, i.e., α-LCT and δ-LIT formed exclusively stable monomers during our screenings, whereas δ-LIT and LCT oligomers were intriguingly observed only on cryoEM grids.

Considering in addition that previous studies suggesting that α-LTX exists mainly in its dimerized or tetramerized form, we conclude that different LaTXs display different oligomerization characteristics. Indeed, although the dimerization interface suggests a general induced fit mechanism in LaTXs, the surface of the involved AR-domain cleft is not highly conserved in the LaTX family (Supplementary Fig. 1 and Supplementary Fig. 6c, g).

The electrophysiological analysis at single channel resolution allowed us to confirm the tetramerization process for both recombinant expressed LaTX samples (precursor δ-LIT and mature α-LCT) in an indirect way, since a complete LaTX tetramer is the prerequisite of functional pore-formation and thus enabling measurements of single channel currents. Indeed, our samples showed full activity and ion-channel gating events of single pore units were detected under the conditions of the high-resolution electrophysiological experiment. This is in complete agreement with previous experiments on α-LTX purified from the venom, which was also shown to form pores on artificial lipid bilayers, but efficient incorporation into biological membranes was only achieved in presence of specific receptors^17–19^. With regard however to our experiments on δ-LIT, we assume that rapid concentration of the sample during cryoEM grid preparation might have been an important factor, for successful assembly of the soluble tetramer, even for a small subpopulation of particles. The charge on the artificial bilayers might be another important factor for efficient LaTX recruitment and subsequent pore formation in the electrophysiology experiments, due to the bipolar charge distribution of the AR-domains. Under physiological conditions, the individual LaTX receptors are however apparently the critical factors for efficient toxin recruitment, assembly of the tetramer and subsequent pore formation^17–19^. Similar dependencies are well known for prepore oligomers of other toxins assembling at the membrane prior pore formation^54^. Oligomerization might also be reinforced by additional factors in the venom of the spider or receptor mediated interactions at the outer cell surface. Latrodectins, low molecular weight proteins characterized from the black widow venom, are known for example to associate to LaTXs and suspected to enhance their potency by altering the local ion balance^55^.

### Implications for latrotoxin receptor recognition, pore-formation, and Ca^2+^ sensing

Neurexin and latrophilin are two well-studied receptors of latrotoxin^36, 56^, but the receptor-binding site (also for their respective invertebrate homologues) on LaTXs is still unknown. On the one hand, the helical bundle, connector and plug domains are buried deeply in the crane claw-like tetramer and the empty space below the helical bundles is most probably required for subsequent pore formation events (Fig. 5a). The four outer claw fingers formed by AR-domains contribute on the other hand the largest exposed surface for receptor interaction. The flexibility of the AR-domain in LaTX might provide a versatile and adaptive platform necessary for sensing and binding three structurally different receptors^57, 58^. Furthermore, the AR-domains of different LaTXs display a low level of conservation, which might be a result of evolution for the purpose of toxin specialization to specific preys. In addition, our data suggest, that the inhibitory domain located at the tail of the AR-domain, probably interrupts the toxin-receptor interface. Therefore, LaTX uses most likely the lower half ARs for receptor recognition. Different LaTXs vary indeed in the number of their ARs, but high-resolution structures of LaTX-receptor complexes are now necessary, towards understanding their specificity in detail.

Interestingly, the helical bundle domain of LaTX is reminiscent of domain I of the pore forming Cry toxin, in which six (but not five) amphipathic helices surround a hydrophobic central helix. Moreover, an α-helix in Cry toxin domain I is also interrupted by a short loop^59, 60^ as we observed in the H7 and H9 helices in our structure. Although the exact pore formation mechanism of Cry toxins is yet unknown, an umbrella model of toxin-insertion, derived from structural studies on colicin, is widely accepted^61^. Recently, the RhopH complex, a pore forming protein of malaria parasites was in addition shown to possess an intriguing similar helical bundle domain to the helical bundle domain of LaTX^62^. This suggests a common strategy in until recently unrelated pore forming proteins, to protect central hydrophobic surface helices prior membrane insertion, but further studies are now required to unravel possible similarities in the respective pore formation mechanisms.

It is known that LaTX pores are permeable for cations and small molecules such as ATP or acetylcholine. At the given ionic conditions the presented electrophysiological measurement further reveal conductance values that are in the order of magnitude as typically observed for K^+^ or Ca^2+^ specific channels with comparable ion concentrations^63, 64^. The LaTX pore is stabilized by Ca^2+^ and has a preferred permeability for this ion, suggesting binding sites in the LaTX pore specialized for Ca^2+^ sensing. A flexible loop in the pore can match both requirements: four loops might form an ion filter-like structure through coordinate bonds with a Ca^2+^, which can further facilitate the intake of the following Ca^2+^. In the absence of Ca^2+^, the loops become disordered, as observed for KcsA in the absence of K^+^ ions in the filter region^65^, but the pore might be then large enough to pass through the other substrates. Aspartic acid (Asp) and glutamic acid (Glu) residues have been known to play a critical role in Ca^2+^ filtering^64, 66^. There are three Asp residues and five Glu residues strictly conserved at the helical bundle domain: Asp232, Asp289, Asp427, Glu121, Glu132, Glu146, Glu185, and Glu203 (residue numbers according to δ-LIT).

### LaTX mechanism of oligomerization prior membrane insertion

Taking all observations together, we propose a four-step mechanism of oligomerization and membrane binding of LaTXs (Fig. 5b). Firstly, the inhibitory domain of LaTX is removed by proteolytic cleavage. This enables the toxins to be recognized by receptors at the extracellular side of the cell membrane. The negatively charged C-terminal tails of AR-domains are further attracted by the cations (e.g. Na^+^ or Ca^2+^) at the extracellular side of the cell membrane, which might be crucial to orientate the molecules properly, with the claw tips directly facing the membrane. LaTXs oligomerize in a cyclic sequential manner, with four protomers rotated 90 degrees relative to each other. In particular, the individual protomers dock into each other, via the insertion of the plug domain of one protomer into the cleft formed by the AR-domain of the adjacent protomer towards the formation of the tetramer. For α-LCT (and possibly also for other LaTXs with longer C-terminal tails of 22 ARs) with protomers in the more compact state due to additional interactions between the helical bundle domain and the longer AR-tail, this interaction is expected to trigger in addition a conformational change for each protomer and stabilize the extended conformation. The resulting tetramer resembles in shape an open crane claw (prepore; membrane insertion competent state). Notably, in this orientation, the bottom part of the cylindrical helical bundle domains, composed each of five parallel aligned helices protecting the central putative transmembrane helix, are exposed, perpendicular aligned and directly facing the membrane. Even though the alternative possibility (i.e., approaching the membrane from the plug domain side) cannot be completely excluded, this scenario is more reasonable considering the bipolar charge of the AR-tail and the overall arrangement of the putative transmembrane domains (Supplementary Fig. 16). This orientation of the soluble prepore towards the membrane, suggests that subsequent pore formation events, might involve massive rearrangements within the four helical bundle domains, resulting, among other events, into downwards injection of the putative transmembrane helices for membrane penetration and finally formation of a transmembrane channel.

## Concluding remarks

Our cryoEM results reveal the general architecture of LaTXs and allow us in combination with first functional studies to understand key steps of LaTX action at molecular level. Future studies of receptor-bound LaTXs in membrane inserted state together with mutational analysis of Ca^2+^ sensing candidate loops and subsequent electrophysiological studies will be necessary to shed light into the intriguing structure and physiological function of the LaTX pore.

## Methods

### Protein expression and purification

Our expression protocol was modified from Volynski K.E., et. al.^8^ based on secretion in the baculovirus expression system. The cDNAs of mature α-LCT (residues 16–1211; UniProtKB Q9XZC0) and full-length δ-LIT (residues 1–1214; UniProtKB Q25338) were optimized for recombinant protein expression in insect cells and fused with an N-terminal honeybee melittin signal peptide (MKFLVNVALVFMVVYISYIY)^67^ to enhance the efficiency of secretion. A Strep-tag II followed by an HRV-3C protease cleavage site was added after the melittin peptide, and a C-terminal His_8_ was added with a thrombin cleavage site. All DNA fragments were synthesized (GenScript Biotech) and cloned into a pACEBac1 plasmid^68^. For producing the mature δ-LIT, Domain I (residues 1–28) was deleted from the construction, and a TEV protease site was inserted between Domain III (CTD) and Domain IV (after residue 1019). The sequences of primers used for deleting Domain I and introducing TEV protease site were shown in Supplementary Table 2. All the final plasmids used in the present study are shown in Source Data.

The bacmids were generated by transforming 200 ng of each plasmid to DH10EMBacY *E.coli* cells. Positive baculovirus genomes were selected using blue/white screening on LB-agar plates containing 100 μg/ml Ampicillin, 10 μg/ml Gentamicin, 10 μg/ml tetracycline, 500 μg/ml 5-Bromo-4-chloro-3-indolyl-β-D-galactopyranoside (X-Gal), and 0.5 mM Isopropyl β-D-1-thiogalactopyranoside (IPTG). The white single colonies were inoculated into 5 ml LB containing 10 μg/ml Gentamicin at 37 °C for 24 h. The *E.coli* cells were lysed by the P1, P2, and N3 buffers of Plasmid Miniprep Kit (QIAGEN), followed by isopropanol precipitation. 700 μl isopropanol was applied to 800 μl cell lysate. The pellets were collected by centrifugation at 16,000 g for 10 min, washed with 200 μl 70% (v/v) ethanol, and solubilized in 50 μl sterilized water. The final concentration of bacmid was approximately 3 μg/μl.

For generating the virus stocks, 10 μg of each bacmid were added with 250 μl Sf-900 II serum-free medium (Thermo Fisher Scientific) and 4 μl of FuGENE HD Transfection Reagent (Promega). The mixtures were transfected into 3 ml of 0.5 × 10^6^ cells/ml Sf9 cells and the supernatants, namely the V_0_ virus stocks, were collected after incubation at 27 °C for 72 h. In order to obtain the virus stocks in higher titration and larger amount, V_1_ and V_2_ virus stocks were generated by suspension culture of the 1.0 × 10^6^ cells/ml Sf9 cells infected with 0.2%(v/v) virus from the previous step at 27 °C in 100 rpm for 72 h and stored at 4 °C with 10% fetal bovine serum (FBS) (Thermo Fisher Scientific). The titration of the final V_2_ virus stocks was approximately 6.0 × 10^8^ PFU/ml measured by plaque assay.

For large scale expression, 2.0 × 10^6^ cells/ml Hi5 cells were infected with the V_2_ virus at a multiplicity of infection (MOI) of 1-2. After suspension culture at 27 °C in 100 rpm for 72 h, the Hi5 cells were removed by centrifugation. The supernatants containing the secreted proteins were chilled to 4 °C and adjusted to pH 8.0 by adding Tris. All the following procedures were conducted at 4 °C unless otherwise noted. White precipitation formed during pH adjustment and was removed by passing it through a 0.45 μm filter (Millipore).

Cleared supernatants were applied to gravity-flow columns filled with 10 ml of Strep-Tactin Sepharose resin (iba) and equilibrated in Wash Buffer (100 mM Tris-HCl pH 8.0, 150 mM NaCl, 1 mM Ethylenediaminetetraacetic acid (EDTA)). Subsequently, the columns were washed with 50 ml (5 CV) of Wash Buffer and eluted with Elution Buffer (100 mM Tris-HCl pH 8.0, 150 mM NaCl, 1 mM EDTA, 2.5 mM desthiobiotin). The elutes were fractionated and confirmed by SDS-PAGE. The fractions containing target proteins were pooled and concentrated to 0.5–5 ml. The protein samples were further purified by size exclusion chromatography (SEC) on Superdex 200 increase 10/300 or Superdex 200 26/60 column (GE Healthcare) equilibrated in Wash Buffer. The final concentration was measured by the Bradford method (Bradford protein assay kit, Bio-Rad) and the purity was confirmed by SDS-PAGE and negative stain electron microscopy.

The mature δ-LIT was generated by incubating the elution of Strep-tag affinity chromatography with 10% (w/w) His-PreScission 3C protease and TEV protease (provided by the Protein Chemistry Facility, Max-Planck Institute for Molecular Physiology) at 4 °C overnight. The product was confirmed by SDS-PAGE followed by SEC as aforementioned.

### Negative-stain-EM analysis

Negative-stain-EM was applied to all the three toxin samples to assess sample quality prior cryoEM analysis. 4 μl of each toxin sample with a concentration of 0.01–0.02 mg/ml were applied to a copper grid covered by a carbon layer and incubated for 2 min at room temperature. The excess protein solution was blotted with filter paper. The grids were washed with 10 μl double-distilled water and 0.75% uranyl formate once each, followed by staining with 10 μl 0.75% uranyl formate for 45 sec. Image acquisition was performed using a JEOL JEM-1400 transmission electron microscope operating at an acceleration voltage of 120 kV. Datasets were acquired with a 4k × 4k CMOS camera F416 (TVIPS) at a magnification of × 80,000. The defocus range was approximately −0.8 μm to −1.8 μm at a pixel size of 1.3 Å/px.

### Sample vitrification and cryoEM data acquisition

For cryoEM sample preparation, 4 μl of protein solution were applied onto a freshly glow-discharged holey carbon grid (QUANTIFOIL R 1.2/1.3 mesh 300) and vitrified using a Vitrobot cryo-plunger (Thermo Fisher Scientific). The plunging condition was optimized by several rounds of screening sessions. The final concentrations of mature α-LCT and full-length δ-LIT solution were 0.6 mg/ml and 0.4 mg/ml, respectively.

Two datasets of the α-LCT were collected with a 300 kV Titan Krios microscope (Thermo Fisher Scientific) equipped with an X-FEG and operated by the software EPU (Thermo Fisher Scientific). One image per hole with defocus of −1.3 to −2.5 μm was collected with the K3 Summit (Gatan) direct electron detector in super-resolution mode at a magnification of 105,000 x and a corresponding pixel size of 0.45 Å/px with a GIF quantum-energy filter set to a filter width of 20 eV. Image stacks with 60 frames were collected with a total exposure time of 3 s and a total dose of 69.1 e/Å^2^. These two datasets were combined and used for the following processing.

Datasets of the δ-LIT were collected with a 300 kV Titan Krios microscope (Thermo Fisher Scientific) equipped with an X-FEG and a Cs corrector and operated by the software EPU (Thermo Fisher Scientific). One image per hole with defocus of −1.3 to −2.4 μm was collected with the K3 Summit (Gatan) direct electron detector in super-resolution mode at a magnification of 81,000 x and a corresponding pixel size of 0.45 Å/px with a GIF quantum-energy filter set to a filter width of 20 eV. Image stacks with 60 frames were collected with a total exposure time of 4 s and a total dose of 78.7 e/Å^2^.

The details of the dataset collection are summarized in Supplementary Table 3.

### Image processing and 3D reconstruction

Image transfer, motion correction, and CTF estimation were performed using Motioncor2 and CTFFIND4 implemented with TranSPHIRE^69^, a recently released software package that allows automated on-the-fly processing for cryoEM. In particular, the motion correction was performed by MotionCor2^70^ to create aligned dose-weighted averages. The super-resolution images were binned twice after motion correction to speed up subsequent processing steps. The non-dose-weighted average micrographs were used for CTF estimation performed by CTFFIND 4.1.10^71^. Outliers were removed based on the estimated defocus values and resolution limits. Further image processing was performed using the software package SPHIRE^72^ unless otherwise noted.

Single particles were automatically picked by crYOLO^73^ based on a manually trained model and extracted with a final window size of 224 × 224 pixels. The 2D classification was performed by ISAC^74^ at a pixel size of 3.1 Å/pixel. The Beautifier tool implemented in SPHIRE was utilized for obtaining refined 2D classes. Classes displaying high-resolution features were selected and combined into a subset. For the α-LCT monomer, an initial 3D model was generated by RVIPER^75^ from a previously collected test dataset and subsequently used for 3D refinement in MERIDIEN^72^ with C1 symmetry. Since the resulting 3D reconstruction showed anisotropic resolution and several low-resolution regions, several rounds of 3D classification were performed by SPHIRE and RELION 3.1^76, 77^ until the compact and flexible intermediate classes were separated from the other conformations. The compact subset containing 376,474 particles was further improved by CTF refinement in RELION 3.1, followed by a final round of 3D refinement in MERIDIEN. The flexible intermediate class only contained 61,526 particles. Therefore, only standard 3D refinement was performed to generate a map for secondary structure fitting. The δ-LIT dimer dataset was processed with the same procedure as the α-LCT monomer until the 2D classification step. The window size was enlarged to 360 × 360 pixels due to the larger particle size. After sorting the monomer, dimer, trimer, and tetramer classes into subsets, a multi-reference classification of the dimer subset was performed in SPHIRE followed by an additional round of 3D classification in RELION 3.1. Eventually, a subset containing 81,192 particles was selected. The initial model for 3D refinement was generated by manually combining two α-LCT monomers, as indicated in the 2D class averages and applying a 15 Å low-pass filter. Subsequent polishing and CTF refinement were performed as described for the α-LCT dataset. The subset of monomer was further divided by 3D classification into two classes containing 70,924 and 41,599 particles. The initial model for 3D refinement was generated by the δ-LIT dimer map.

The final half-maps were combined upon masking using a 3D mask generated by the PostRefiner tool implemented in SPHIRE, which in addition automatically determines the B-factor and filters the resulting volume to estimated resolutions. The global resolutions and the 3DFSC were calculated at the gold standard 0.143 criterion using the 3DFSC server^78^. The angular distribution and the local resolution distribution of the final map were analyzed using the o angular_distribution and sp_locres in SPHIRE

### Model building, validation, and visualization

The de novo model building of the compact form of the α-LCT monomer started from a 3D structure prediction using trRossetta (Yang 2020). The resulting model did not agree well with the density maps, therefore it was fragmented into several structural domains according to the secondary structure prediction from RaptorX Property^79^. Rigid-body fitting of the individual domain was then performed using Pymol (The PyMOL Molecular Graphics System, Version 2.0 Schrödinger, LLC). Further refinement was conducted using the real-time molecular-dynamics simulation-based program ISOLDE^80^ implemented in the visualization softwares UCSF Chimera and UCSF ChimeraX^81, 82^, and the resulting model was further refined using a combination of the model editing software COOT^83^ and real-space refinement in Phenix^84^. The above adjustments were performed for several rounds until the model sufficiently fitinto the map. The model of the flexible intermediate state was generated by truncating side chains with the Chainsaw tool in CCP4^85^ and fitted into the corresponding low-resolution map. The starting model of the δ-LIT dimer was generated by the homology-modeling service SWISS-MODEL^86^ from the α-LCT model, which was subsequently fitted into the map using ISOLDE, COOT, and Phenix. The disordered regions and side-chain atoms beyond Cβ in regions where side-chain density was only rarely evident were deleted during the model fitting. Rigid-body fitting into the low-resolution maps of this study (e.g., the flexible intermediate state of α-LCT monomer and the two δ-LIT monomers) was performed with the molecular model of the compact state of α-LCT monomer (4 Å) for α-LCT maps and the protomer A of δ-LIT dimer (4.6 Å) for δ-LIT maps. Fitting was performed with Chimera and further refined using ISOLDE. Further model refinement was not applied due to the limited quality of the maps.

To calculate the protein properties, i.e., surface electrostatics, hydrophobicities, residue conservations, and transmembrane region predictions, the side chains were put back with ideal rotamers (Supplementary Fig. 6). The figures were prepared using UCSF Chimera and ChimeraX^81, 82^. The threshold values (sigma) were calculated by dividing the corresponding contour level by the root-mean-square (RMS) value. Multiple sequence alignment and the prediction of transmembrane helices were done using Clustal Omega^87^ and TMHMM server v2.0^88^, respectively. The surface electrostatics was calculated in APBS^89^ and visualized by UCSF ChimeraX. These sequence features were illustrated by ESPript3^90^ and Adobe Illustrator.

### Electrophysiology

#### Single channel recordings from planar lipid bilayers and data analysis

Planar lipid bilayer measurements using the Compact bilayer platform (Ionovation GmbH) were performed as described in detail previously^91^. In brief: If not explicitly stated otherwise, symmetric conditions (250 mM KCl, 10 mM HEPES, pH 7.0) were used in *cis* and *trans* compartments. The denomination *cis* and *trans* corresponds to the half-chambers of the bilayer unit. Reported membrane potentials are always referred to the *trans* compartment. Bilayer fabrication was performed on PFTE film at a 100 µm prepainted (1 % hexadecane in n-hexane) aperture with a Phosphatidylcholine (18:1) (PC)/Phosphatidylethanolamine (18:1) (PE) (7:3 ratio) lipid mixture (both lipids were purchased from Avanti Polar Lipids) in *n*-pentane using the thinning method^91^. Stock solutions of purified LaTXs with typically 0.5–2.5 mg/ml contained in a buffer of 100 mM NaCl, 20 mM Hepes, pH 7.4 were added to the *cis* compartment under slight stirring to a final protein concentration of ~1 μg/ml to 10 ng/ml.

Ion-channel currents were recorded using an EPC 10 USB amplifier (HEKA Elektronik GmbH) in combination with the Patchmaster data acquisition software (HEKA Elektronik GmbH). For data acquisition a sampling rate of 5 kHz (voltage ramps) and 10 kHz (continuous recording) was used and the data were further analyzed using the Origin package (Origin Lab) and the MATLAB (MathWorks) based Ion-channel-Master software developed in our laboratory^91^.

### The GHK approach

The Goldman-Hodgkin-Katz approach is by far the most commonly used framework to describe ion permeability and selectivity of membranes^47, 92^. Besides the principal difficulties underlying the macroscopic GHK constant field theory, which assumes independent movement of the ions through membrane pores (see references^93–95^ for a detailed discussion) it has been demonstrated that the methodology can be used to obtain reliable semi-quantitative measures for permeation of charged drugs through membranes^48^.

We were interested in obtaining information on the selectivity of the membrane reconstituted δ-LIT. To characterize the ion fluxes mediated by the δ-LIT we employed the following experimental conditions for bilayer containing an unknown number of open δ-LIT channels.

*Permeability δ-LIT:*

1 $${P}_{{{{{{{\rm{K}}}}}}}^{+}}=1.47$$

2 $${P}_{{{{{{{{\rm{Cl}}}}}}}}^{-}}={{{{{{\rm{variable}}}}}}}\left(+{V}_{{{{{{{\rm{cmd}}}}}}}}\right)$$

3 $${P}_{{{{{{{{\rm{Ca}}}}}}}}^{2+}}={{{{{{\rm{variable}}}}}}}\left(-{V}_{{{{{{{\rm{cmd}}}}}}}}\right)* ({{{{{\rm{negative}}}}}}{V}_{{{{{{{\rm{cmd}}}}}}}})$$

*Cation:*

4 $${{z}_{{{{{{{\rm{K}}}}}}}^{+}}=1{{{{{\rm{;}}}}}\;}c}_{{{{{{{\rm{K}}}}}}}^{+}{cis}}=250{{{{{{\rm{mM}}}}}}}{{{{{\rm{;}}}}}}\;{c}_{{{{{{{\rm{K}}}}}}}^{+}{trans}}=25{{{{{{\rm{mM}}}}}}}$$

*Anion:*

5 $${{z}_{{{{{{{{\rm{Cl}}}}}}}}^{-}}=-1.0\;{{{{{\rm{;}}}}}}c}_{{{{{{{{\rm{Cl}}}}}}}}^{-}{cis}}=250{{{{{{\rm{mM}}}}}}}{{{{{\rm{;}}}}}}\;{c}_{{{{{{{{\rm{Cl}}}}}}}}^{-}{trans}}=25{{{{{{\rm{mM}}}}}}}$$

*Calcium:*

6 $${{z}_{{{{{{{{\rm{Ca}}}}}}}}^{2+}}=2\;{{{{{\rm{;}}}}}}c}_{{{{{{{{\rm{Ca}}}}}}}}^{2+}{cis}}=5{{{{{{\rm{mM}}}}}}}{{{{{\rm{;}}}}}}\;{c}_{{{{{{{{\rm{Ca}}}}}}}}^{2+}{trans}} < 10\;\mu {{{{{{\rm{mM}}}}}}}$$

*Zero-current potential:*

*Ca* ^*2+*^ *:*

7 $${V}_{{{{{{{\rm{rev}}}}}}}}\approx 47\pm 2.3{{{{{{\rm{mV}}}}}}}\;{{{{{\rm{experimentalvalue}}}}}}({{\pm }}{{{{{\rm{SD}}}}}})$$

*Cl* ^*1-*^ *:*

8 $${V}_{{{{{{{\rm{rev}}}}}}}}\approx -57\pm 3.4{{{{{{\rm{mV}}}}}}}\;{{{{{\rm{experimentalvalue}}}}}}({{\pm }}{{{{{\rm{SD}}}}}})$$

Using these values (1–6), the above concentrations in the *cis* and *trans* compartment and considering that the assumptions of the GHK-theory are valid under the applied conditions we can use Eqs. (9–13) to calculate the expected current–voltage relation for the above bilayer membrane containing an unknown number of active δ-LIT channels.

Extrapolating the current slope at high positive and negative voltages linearly to zero net current (Fig. 4b) yields a reversal potential of ≈ 47 mV (7) and ≈ −57 mV (8), these values would be compatible with a very high calcium or chloride selectivity of the mature δ-LIT channel depending on the current direction (${{P}_{{{{{{{{\rm{Ca}}}}}}}}^{2+}}/P}_{{{{{{{\rm{K}}}}}}}^{+}}/{P}_{{{{{{{{\rm{Cl}}}}}}}}^{-}}\cong 400/1.47/1$) flux of calcium (−*V*_mem_) or $({{P}_{{{{{{{{\rm{Ca}}}}}}}}^{2+}}/P}_{{{{{{{\rm{K}}}}}}}^{+}}/{P}_{{{{{{{{\rm{Cl}}}}}}}}^{-}}\cong 1/1.47/150)$ chloride currents (*V*_mem_)^93–95^. In summary, the course of the current–voltage relation in the lower panel of Fig. 4d can be explained from our calculations if the currents at negative *V*_cmd_ are carried from *cis* to *trans* mainly by Ca^2+^ ions and at positive *V*_cmd_ predominantly by Cl^−^ ions.

*GHK-current equations*:

9 $${I}_{x}\left(V,{P}_{X},z,{c}_{{cis}},{c}_{{trans}}\right)={{P}_{x}z}^{2}\frac{V{F}^{2}}{{RT}}{{\cdot }}\frac{\left({c}_{x,{cis}}-{c}_{x,{trans}}{\exp }\left(\frac{-{zFV}}{{RT}}\right)\right)\,}{1-{\exp }\left(\frac{-{zFV}}{{RT}}\right)}$$

10 $${I}_{{{{{{{\rm{K}}}}}}}^{+}}\left(V\right)=I\left(V,{P}_{{{{{{{\rm{K}}}}}}}^{+}},{z}_{{{{{{{\rm{k}}}}}}}^{+}},{c}_{{{{{{{\rm{K}}}}}}}^{+}{cis}},{c}_{{{{{{{\rm{K}}}}}}}^{+}{trans}}\right),$$

11 $${I}_{{{{{{{{\rm{Cl}}}}}}}}^{-}}\left(V\right)=I\left(V,{P}_{{{{{{{{\rm{Cl}}}}}}}}^{-}},{z}_{{{{{{{{\rm{Cl}}}}}}}}^{-}},{c}_{{{{{{{{\rm{Cl}}}}}}}}^{-}{cis}},{c}_{{{{{{{{\rm{Cl}}}}}}}}^{-}{trans}}\right)$$

12 $${I}_{{{{{{{{\rm{Ca}}}}}}}}^{2+}}\left(V\right)=I\left(V,{P}_{{{{{{{{\rm{Ca}}}}}}}}^{2+}},{z}_{{{{{{{{\rm{Ca}}}}}}}}^{2+}},{c}_{{{{{{{{\rm{Ca}}}}}}}}^{2+}{cis}},{c}_{{{{{{{{\rm{Ca}}}}}}}}^{2+}{trans}}\right)$$

13 $$\sum I\left(V\right)={I}_{{{{{{{\rm{K}}}}}}}^{+}}\left(V\right)+{I}_{{{{{{{{\rm{Cl}}}}}}}}^{-}}\left(V\right)++{I}_{{{{{{{{\rm{Ca}}}}}}}}^{2+}}\left(V\right)$$

Using Eqs. (9–13) we calculated the corresponding current–voltage relations (Fig. 4e) for the ionic conditions given above using a Mathcad (PTC-Software) based routine^46^.

### Reporting summary

Further information on research design is available in the Nature Research Reporting Summary linked to this article.

## Supplementary information

Supplementary Information

Description of Additional Supplementary Files

Supplementary Movie 1

Supplementary Movie 2

Supplementary Movie 3

Reporting summary

## Data Availability

The cryoEM datasets of α-LCT and δ-LIT generated in this study have been deposited in EMPIAR under accession codes EMPIAR-10827 and EMPIAR-10828. The cryoEM maps of α-LCT monomer and δ-LIT dimer have been deposited in the Electron Microscopy Data Bank (EMDB) under accession codes EMD-13642 and EMD-13643. The coordinates of the corresponding models have been deposited to the Protein Data Bank (PDB) under accession codes 7PTX and 7PTY. The full sequences of the plasmids used in this study and the raw electrophysiological data as tab-separated asci-files of the respective individual figures are provided in the Source data. Other data are available from the corresponding author upon reasonable request. Source data are provided with this paper.

## Source data

Source Data
