# Supplementary material for: Molecular architecture of black widow spider neurotoxins
Source: Nat Commun. 2021 Nov 29;12:6956. doi: 10.1038/s41467-021-26562-8 (PMC8630228; doi:10.1038/s41467-021-26562-8)
Supplement: Supplementary file 1 — Supplementary Information [file 41467_2021_26562_MOESM1_ESM.pdf]

Supplementary Information

of

Molecular architecture of black widow spider neurotoxins

Minghao Chen, Daniel Blum, Lena Engelhard, Stefan Raunser, Richard Wagner and

Christos Gatsogiannis

Contents:

1. Supplementary Figures
2. Supplementary Tables
3. Supplementary References

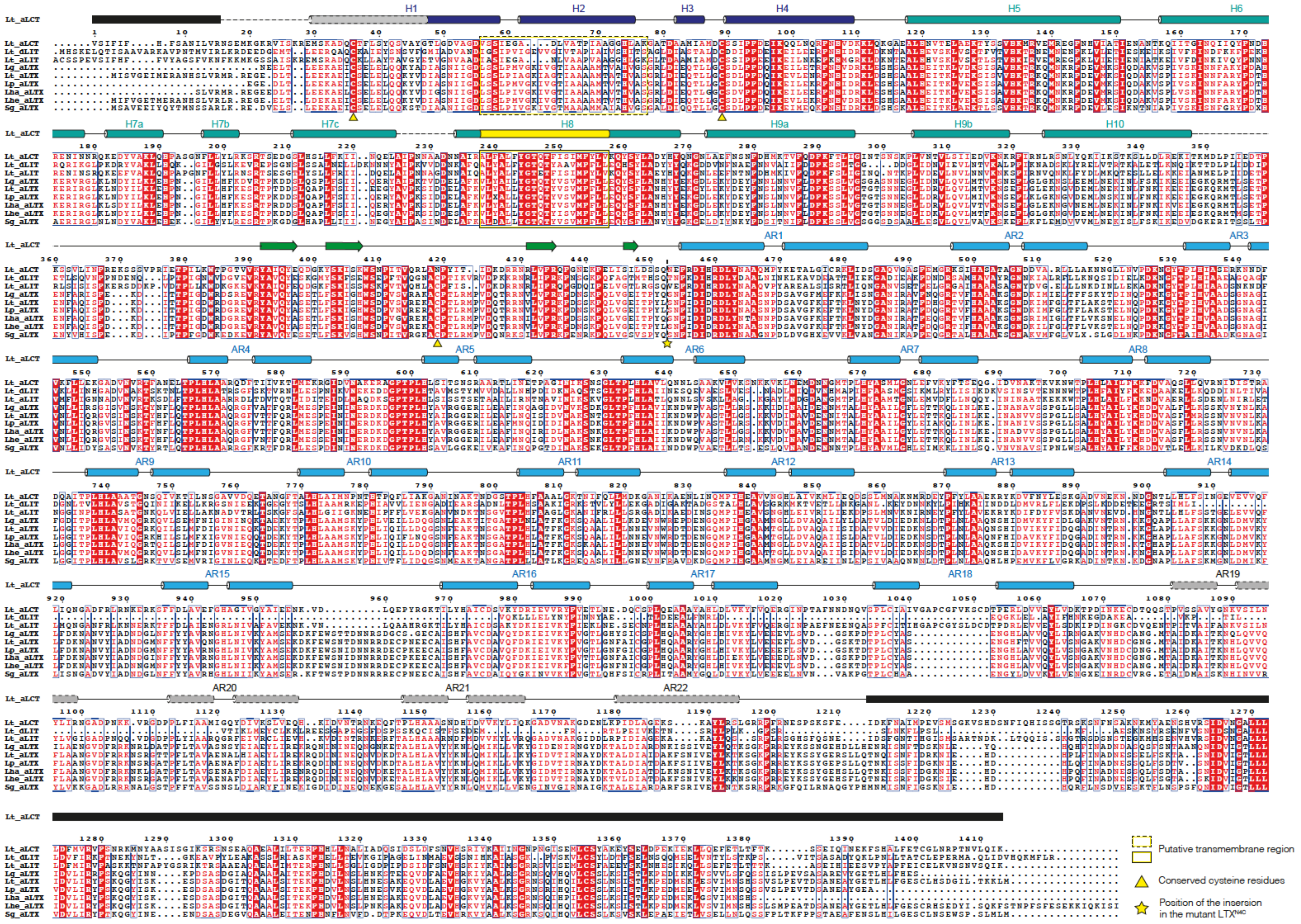

Lt_aLCT
Lt_dLIT
Lt_aLIT
Lg_aLTX
Lt_aLTX
Lp_aLTX
Lha_aLTX
Lhe_aLTX
Sg_aLTX
H1
H2
H3
H4
H5
H6
H7a
H7b
H7c
H8
H9a
H9b
H10
AR1
AR2
AR3
AR4
AR5
AR6
AR7
AR8
AR9
AR10
AR11
AR12
AR13
AR14
AR15
AR16
AR17
AR18
AR19
AR20
AR21
AR22
Putative transmembrane region
Conserved cysteine residues
Position of the insertion in the mutant LTX^N4C

Supplementary Figure 1. **Sequence alignment of latrotoxin protein sequences.** Lt_αLCT, α-latrocrustatoxin from *Latrodectus tredecimguttatus* (Mediterranean black widow), UniprotKB ID: Q9XZC0 (LCTA_LATTR), same below. Lt_δLIT, δ-latroinsectotoxin from *L. tredecimguttatus,* Q25338 (LITD_LATTR). Lt_αLIT, α-latroinsectotoxin from *L. tredecimguttatus,* Q02989 (LITA_LATTR). Lg_αLTX, α-latrotoxin from *Latrodectus geometricus* (brown widow), L7XCU0 (L7XCU0_LATGE). Lt_αLTX, α-latrotoxin from *L. tredecimguttatus,* P23631 (LATA_LATTR). Lp_αLTX, α-latrotoxin from *Latrodectus pallidus* (white widow spider), L7XDS4 (L7XDS4_9ARAC). Lha_αLTX, α-latrotoxin from *Latrodectus hasselti* (redback spider), G0LXV8 (LATA_LATHA). Lhe_αLTX, α-latrotoxin from *Latrodectus hesperus* (western black widow), P0DJE3 (LATA_LATHE). Sg_αLTX, α-latrotoxin from *Steatoda grossa* (brown house spider), L7X8P2 (L7X8P2_STEGR). The secondary structure of α-LCT is shown at the top. Dashed lines or black boxes indicate disorder and/or flexible regions not included in the α-LCT model.

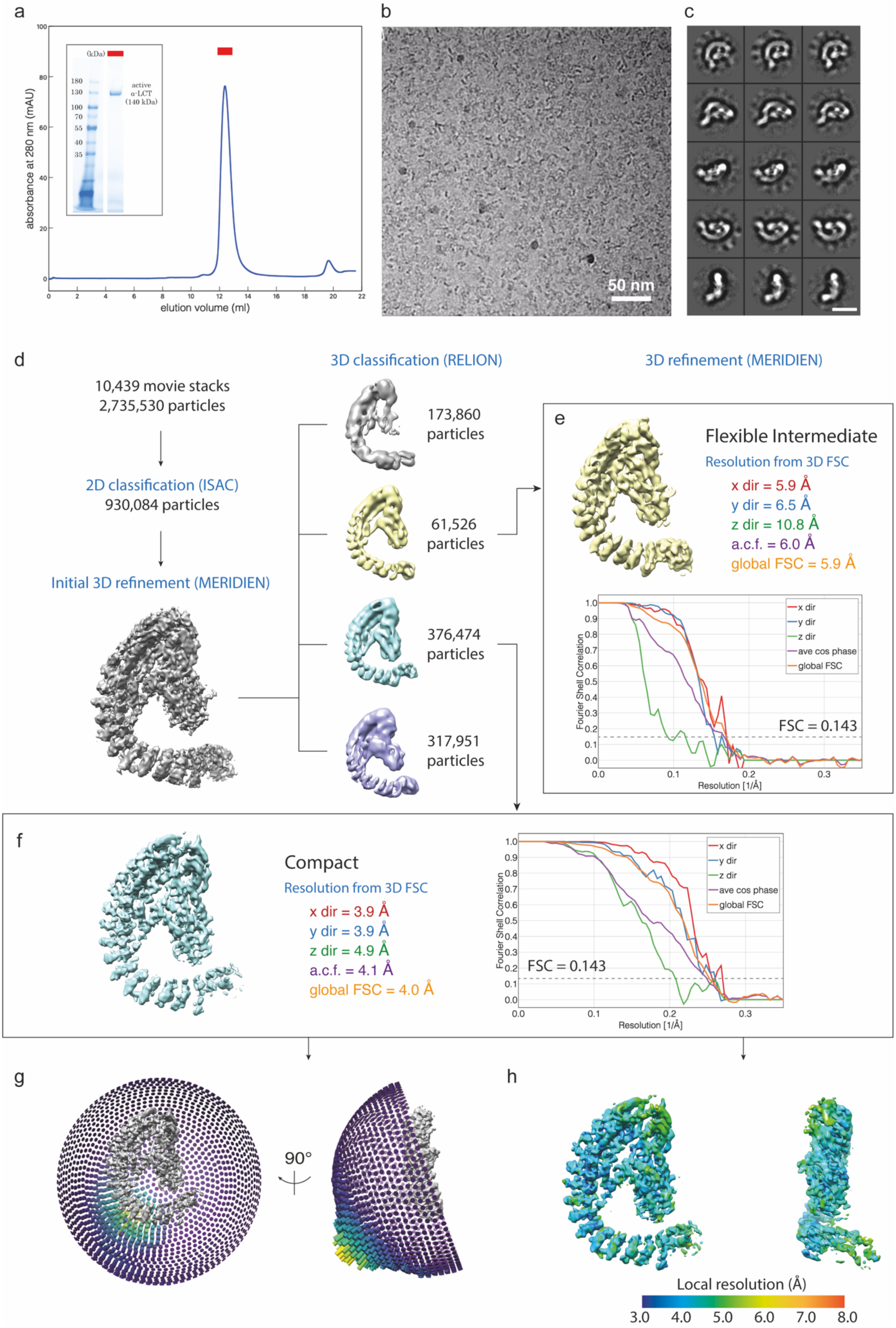
a
absorbance at 280 nm (mAU)
elution volume (ml)
(kDa)
active α-LCT (140 kDa)
b
50 nm
c
d
10,439 movie stacks
2,735,530 particles
2D classification (ISAC)
930,084 particles
Initial 3D refinement (MERIDIEN)
3D classification (RELION)
173,860 particles
61,526 particles
376,474 particles
317,951 particles
3D refinement (MERIDIEN)
e
Flexible Intermediate
Resolution from 3D FSC
x dir = 5.9 Å
y dir = 6.5 Å
z dir = 10.8 Å
a.c.f. = 6.0 Å
global FSC = 5.9 Å
Fourier Shell Correlation
Resolution [1/Å]
ave cos phase
FSC = 0.143
f
Compact
Resolution from 3D FSC
x dir = 3.9 Å
y dir = 3.9 Å
z dir = 4.9 Å
a.c.f. = 4.1 Å
global FSC = 4.0 Å
g
90°
h
Local resolution (Å)
3.0 4.0 5.0 6.0 7.0 8.0

Supplementary Figure 2. **CryoEM sample preparation, image acquisition, and data processing of mature, truncated α-LCT. a**, Size exclusion chromatography (SEC) profile of α-LCT, showing only one peak corresponding to the monomer. The inset shows an SDS-PAGE of the peak (red bar). A total of n=9 equivalent experiment have been conducted. **b**, A representative cryoEM micrograph of α-LCT. A total of n=10,439 equivalent images have been collected. **c**, Representative reference-free 2D class averages. Scale bar: 10 nm **d**, Flowchart of α-LCT cryoEM data processing workflow. See 'data processing' section in Methods for details. **e, f**, 3D FSCs and final unprocessed EM maps of a flexible intermediate (e) and the best resolved compact conformation (f) of α-LCT monomer, respectively. **g**, Angular distribution for the final reconstruction of compact"α-LCT. **h**, EM map of compact α-LCT colored by local resolution.

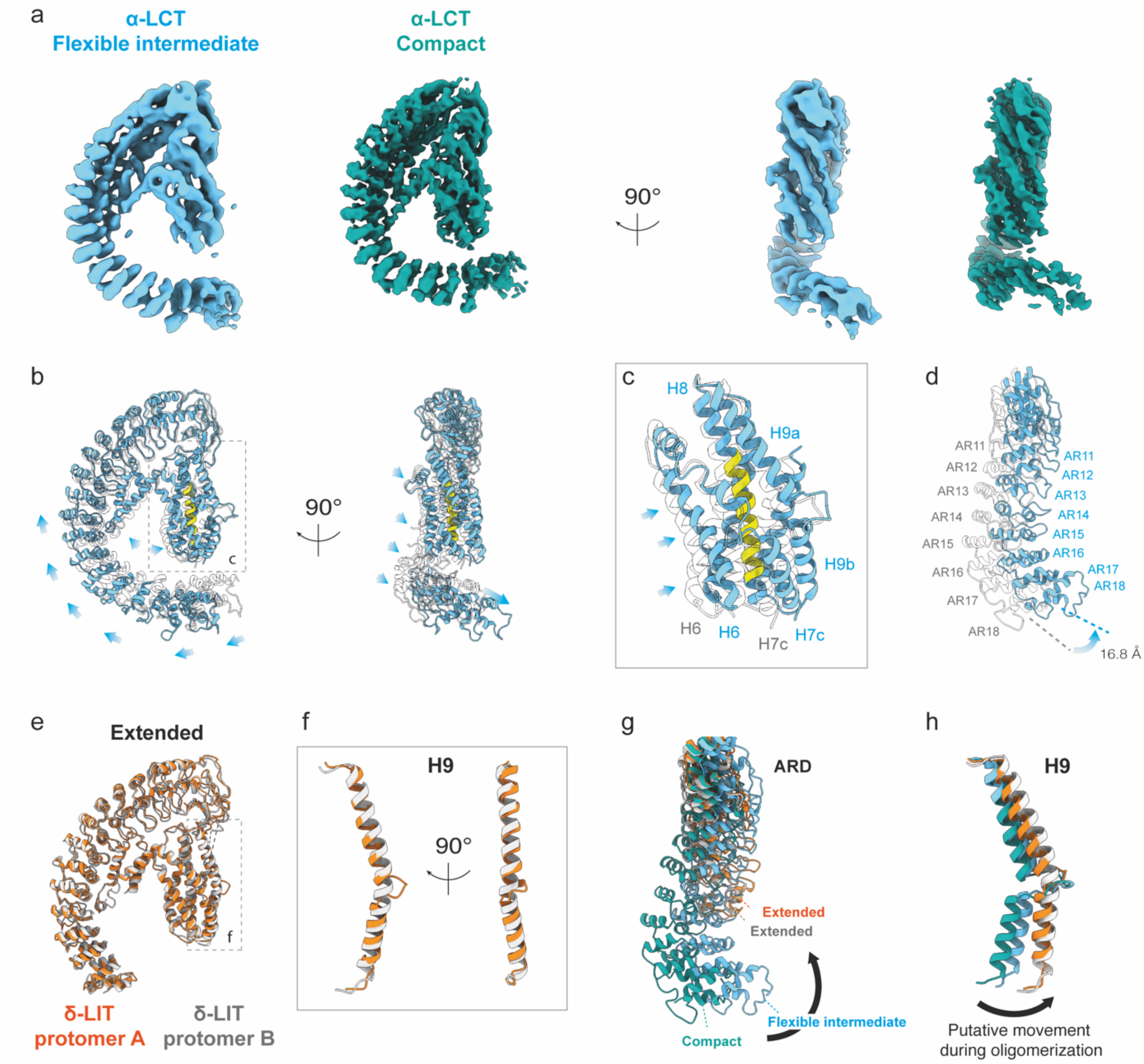

Supplementary Figure 3. **Different conformations of LaTX protomers. a**, Side by side comparison of the EM maps of the α-LCT monomer in the intermediate (cyan) contoured at 7σ and compact conformation (sea green) contoured at 16σ. **b**, Molecular model of the α-LCT intermediate superimposed with the compact conformation (transparent). Arrows indicate domain movement from the compact to the flexible intermediate intermediate conformation. **c**, **d**, Magnified view of the helical bundle domains (c) and the terminal ARs (d) 11-18. **e**, Comparison of the two protomers of the δ-LIT dimer. Protomer A: orange; Protomer B: gray. **f**, Magnified view of the helix H9. **g**,**h**, Comparison of the AR-domains (ARD) and helix H9 of all four conformations, respectively.

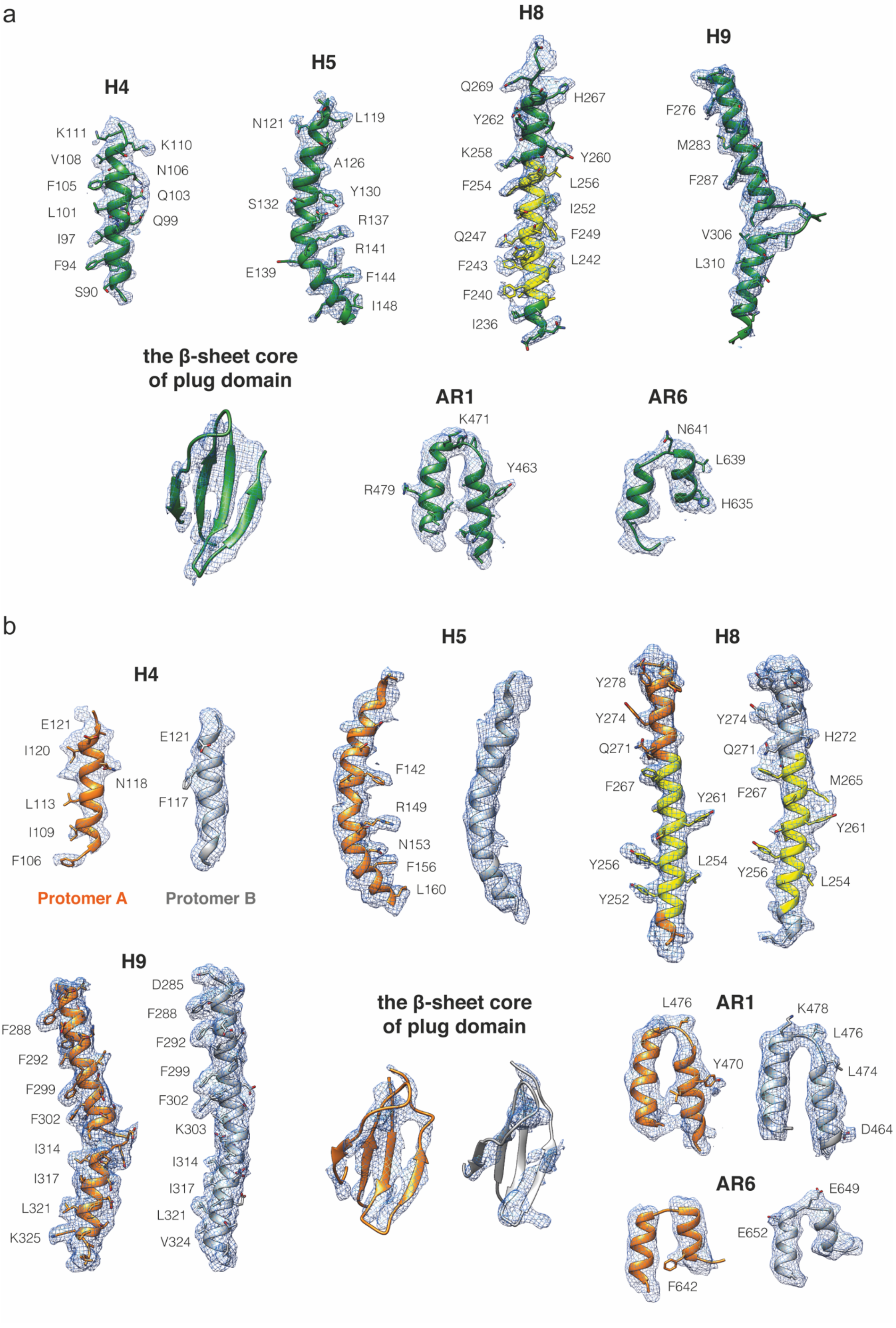

Supplementary Figure 4. CryoEM densities of α-LCT and δ-LIT. Superposition of segments of the molecular model of α-LCT(a) and δ-LIT (b) with the cryoEM densities. The maps are shown as blue mesh with contour level of 13σ and 10σ for α-LCT and δ-LIT, respectively.

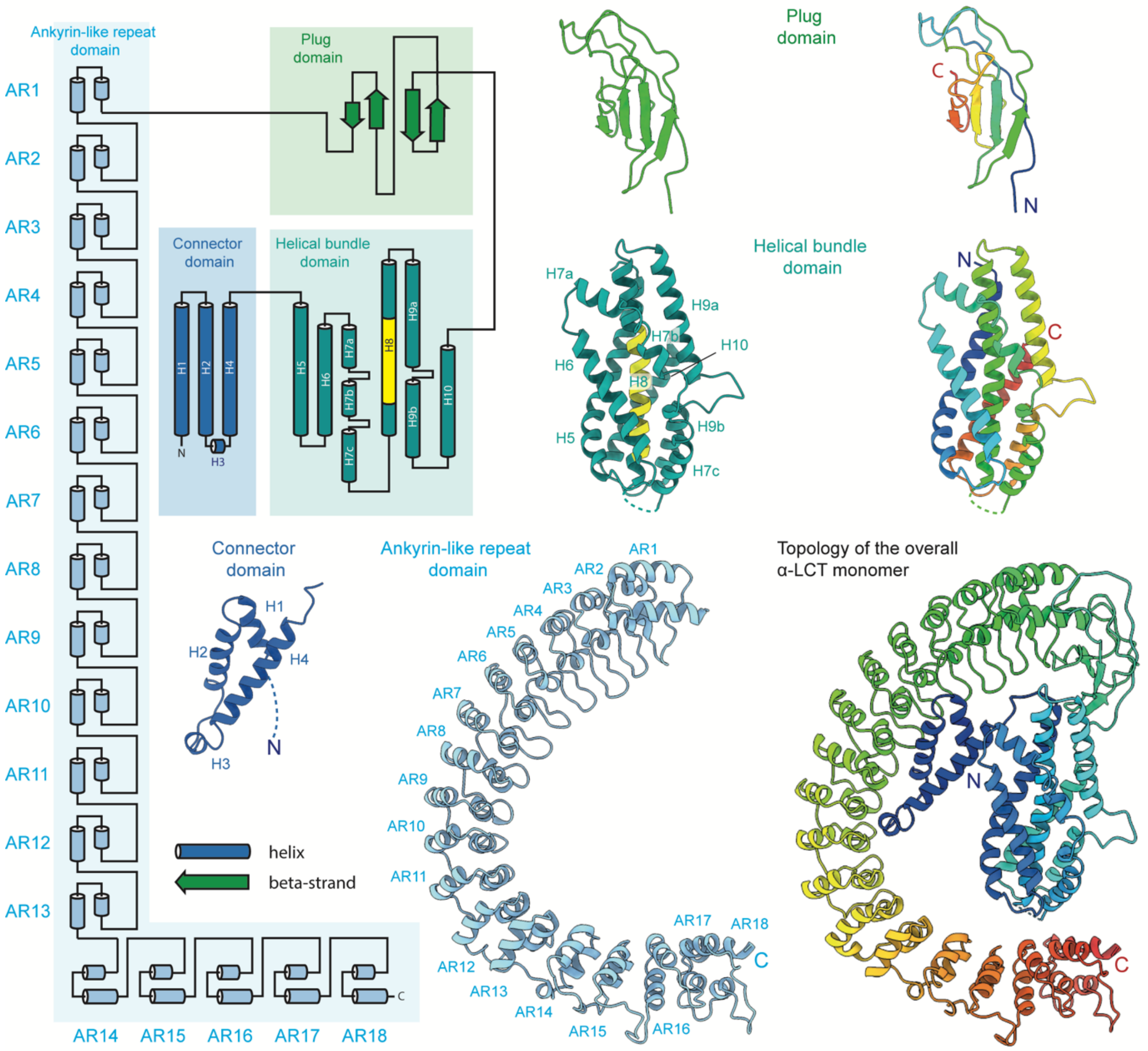

Supplementary Figure 5. **Topology diagram of α-LCT.** The connector domain (blue), helical bundle domain (sea green), transmembrane region (yellow), plug domain (green) and ankyrin-like repeat domain (cyan) are shown together with the respective molecular models. The models of plug domain, helical bundle domain, and the overall α-LCT monomer are colored with a rainbow spectrum (N-terminal blue; C-terminal red).

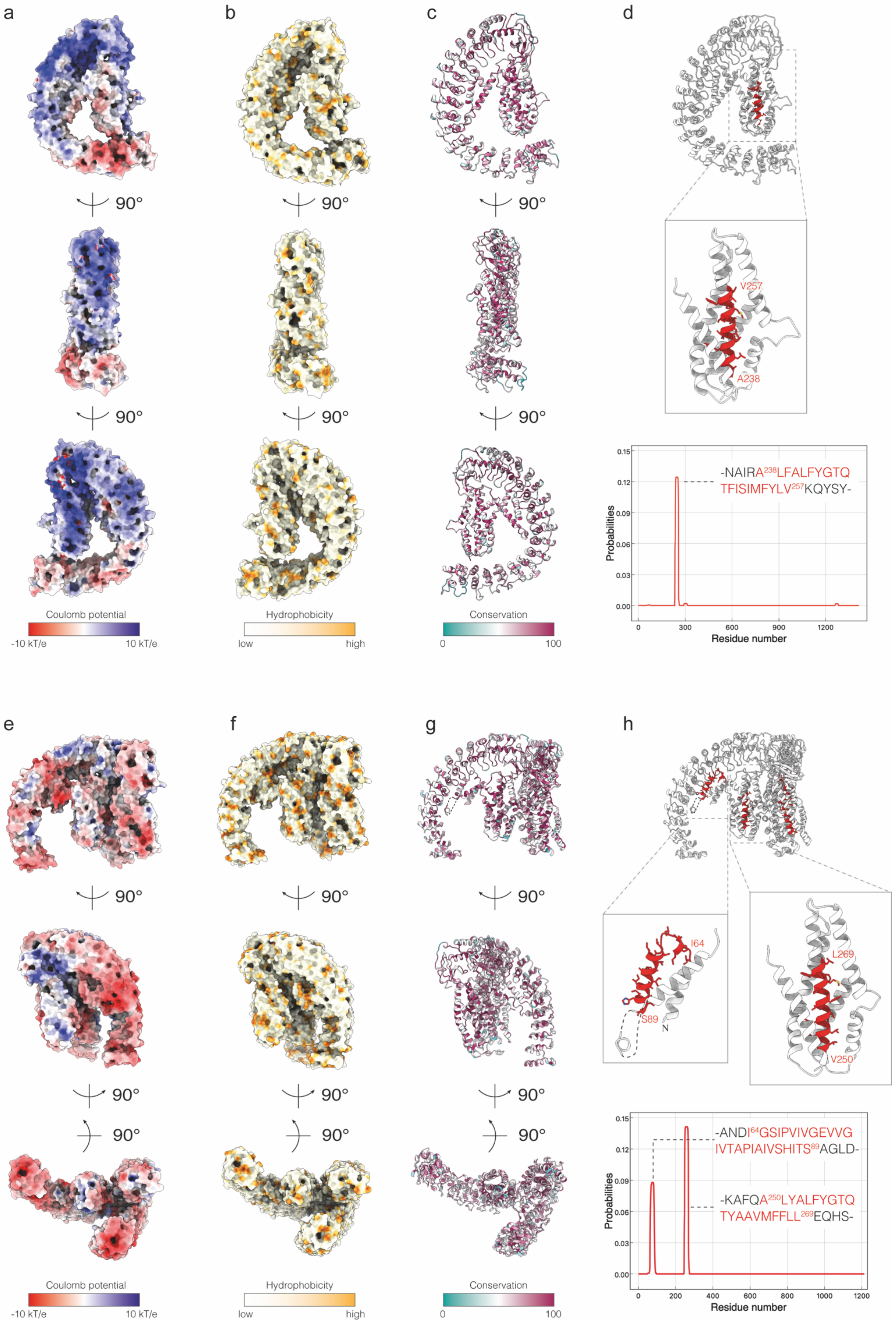

a
b
c
d
90°
V257
A238
Coulomb potential
-10 kT/e
10 kT/e
Hydrophobicity
low
high
Conservation
0
100
-NAIRA238LFALFYGTQ
TFISIMFYLV257KQYSY-
Probabilities
Residue number
e
f
g
h
I64
S89
N
L269
V250
-ANDI64GSIPVIVGEVVG
IVTAPIAIVSHITS89AGLD-
-KAFQA250LYALFYGTQ
TYAAVMFFLL269EQHS-

Supplementary Figure 6. **Comparative analysis of the biophysical properties and topology of the predicted transmembrane helices a**, Surface electrostatics of the α-LCT monomer at pH 7.2 calculated in APBS. Red: -10 kT/e; Blue: +10 kT/e. **b**, Surface hydrophobicity. White: low; Yellow: high **c**, Amino acid conservation. Maroon: fully conserved positions; Cyan: no conservation. **d**, The putative transmembrane region (red) of α-LCT predicted by TMHMM Server v. 2.0. **e-h**, The same structural analysis performed for the δ-LIT protomer extracted from the molecular model of the dimer. Notice there are two transmembrane regions predicted from the δ-LIT sequence.

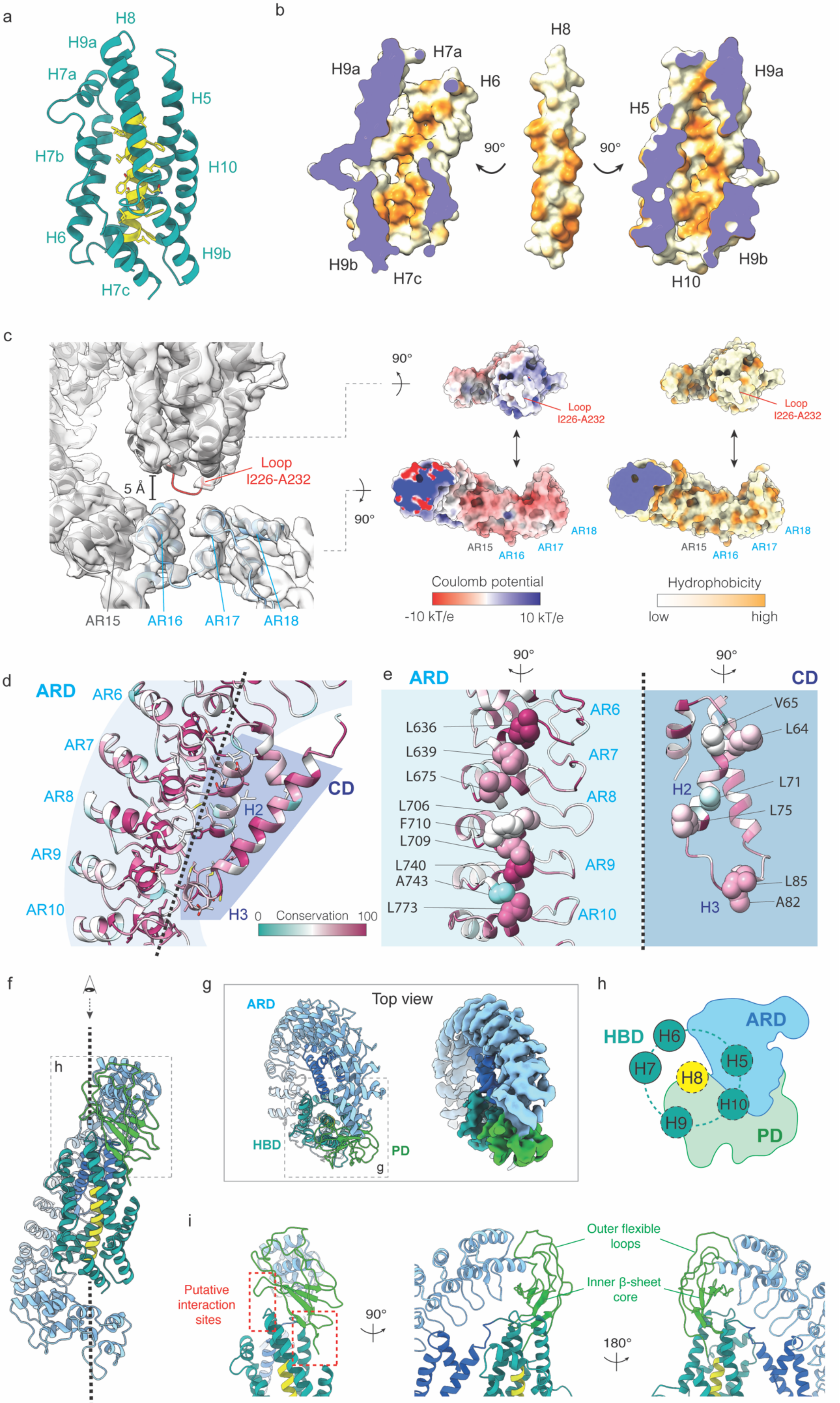
a
H8
H9a
H7a
H5
H7b
H10
H6
H9b
H7c
b
H7a
H9a
H6
H8
H9a
H5
90°
90°
H9b
H7c
H9b
H10
c
90°
Loop
I226-A232
Loop
I226-A232
Loop
I226-A232
5 Å
90°
AR18
AR18
AR15
AR16
AR17
AR15
AR16
AR17
AR15
AR16
AR17
AR18
Coulomb potential
-10 kT/e
10 kT/e
Hydrophobicity
low
high
d
ARD
AR6
AR7
AR8
AR9
AR10
CD
H2
H3
0
Conservation
100
e
ARD
90°
90°
CD
AR6
L636
L639
AR7
L675
AR8
L706
F710
L709
L740
A743
AR9
L773
AR10
V65
L64
H2
L71
L75
L85
H3
A82
f
h
g
Top view
ARD
HBD
PD
g
h
HBD
H6
ARD
H7
H5
H8
H10
H9
PD
i
Outer flexible
loops
Inner β-sheet
core
Putative
interaction
sites
90°
180°

Supplementary Figure 7. **Structural characteristics of α-LCT a,** Close-up view of the HBD. Helix H9 is shown in the front. **b**, The hydrophobic pocket formed by the HDB. The center helix H8 is shown in the same orientation as **a**, together with sliced views of the pocket formed by the outer helices of the bundle, displaying the hydrophobic surface potential. White: low; Yellow: high. **c,** Surface electrostatics and hydrophobicity of the interface between the HBD and ARD. The close-up view of the interface is shown by a model (red: missing loop between the H7 and H8; blue: AR16-18) superposed with the EM map contoured at 10σ. The electrostatic potential was calculated at pH 7.2 in APBS. Red: -10 kT/e; Blue: +10 kT/e, and the hydrophobicity was calculated in ChimeraX. White: low; Yellow: high **d,** Close-up view of the CD-ARD interface. Ribbons are colored by conservation. **e**, Side views of the interface between ARD and CD. ARD and CD are rotated 90 degrees to the left and right, respectively. The residues which are considered to contribute to the hydrophobic interaction are shown as spheres. **f,g**, Side (e) and top (f) view of α-LCT monomer. The center helix (H8) of HBD in (e) is aligned to the vertical axis. The EM map showed in **f** is contoured at 16σ. **h**, A schematic diagram of the HBD-ARD-PD interface. The upper side of HBD is partially covered by the PD and ARD. **i**, Close-up view of the PD. The putative interaction sites with HBD (left) and the overall two-layer structure of PD , i.e., the flexible loops and the β-sheet core, are indicated. PD:plug domain; HBD:helical bundle domain; CD: connector domain; ARD: ankyrin-like repeat domain

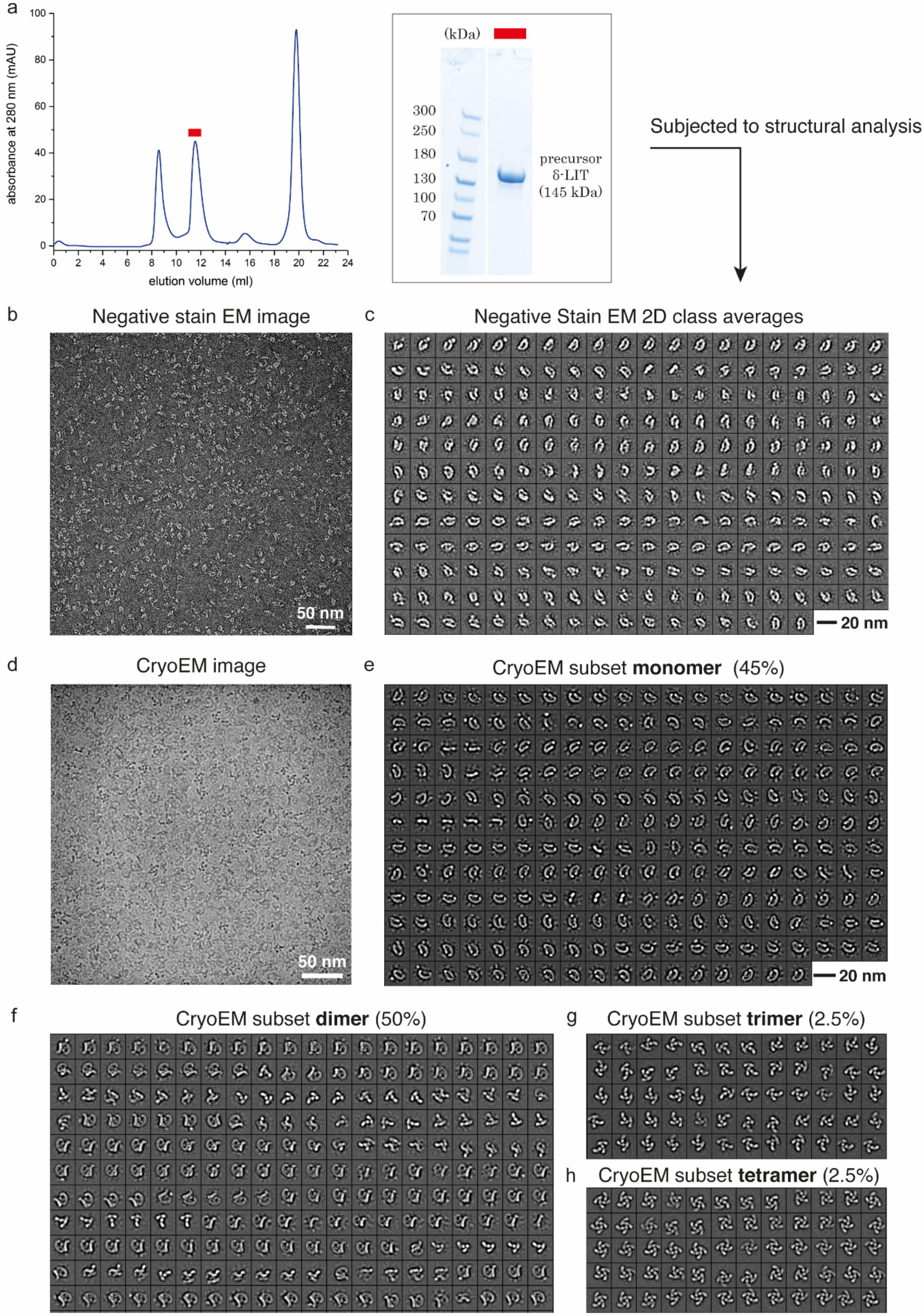
a
absorbance at 280 nm (mAU)
elution volume (ml)
(kDa)
300
250
180
130
100
70
precursor
δ-LIT
(145 kDa)
Subjected to structural analysis
b
Negative stain EM image
50 nm
c
Negative Stain EM 2D class averages
20 nm
d
CryoEM image
50 nm
e
CryoEM subset **monomer** (45%)
20 nm
f
CryoEM subset **dimer** (50%)
g
CryoEM subset **trimer** (2.5%)
h
CryoEM subset **tetramer** (2.5%)

Supplementary Figure 8. Sample preparation, image acquisition, and data processing of the δ-LIT dataset (precursor, full-length). a, Size exclusion chromatography (SEC) profile of δ-LIT. The sample subjected to subsequent cryoEM analysis is indicated (red bar) and confirmed with SDS-PAGE. A total of n=3 equivalent experiment have been conducted. **b**, A representative negative stain micrograph of δ-LIT. A total of n=100 equivalent images have been collected. **c**, Representative reference free 2D class averages. **d**, A representative cryoEM micrograph of δ-LIT. A total of n=2,337 equivalent images have been collected. **e-h**, Representative reference free 2D class averages of monomer (e), dimer (f), trimer (g), and tetramer (h).

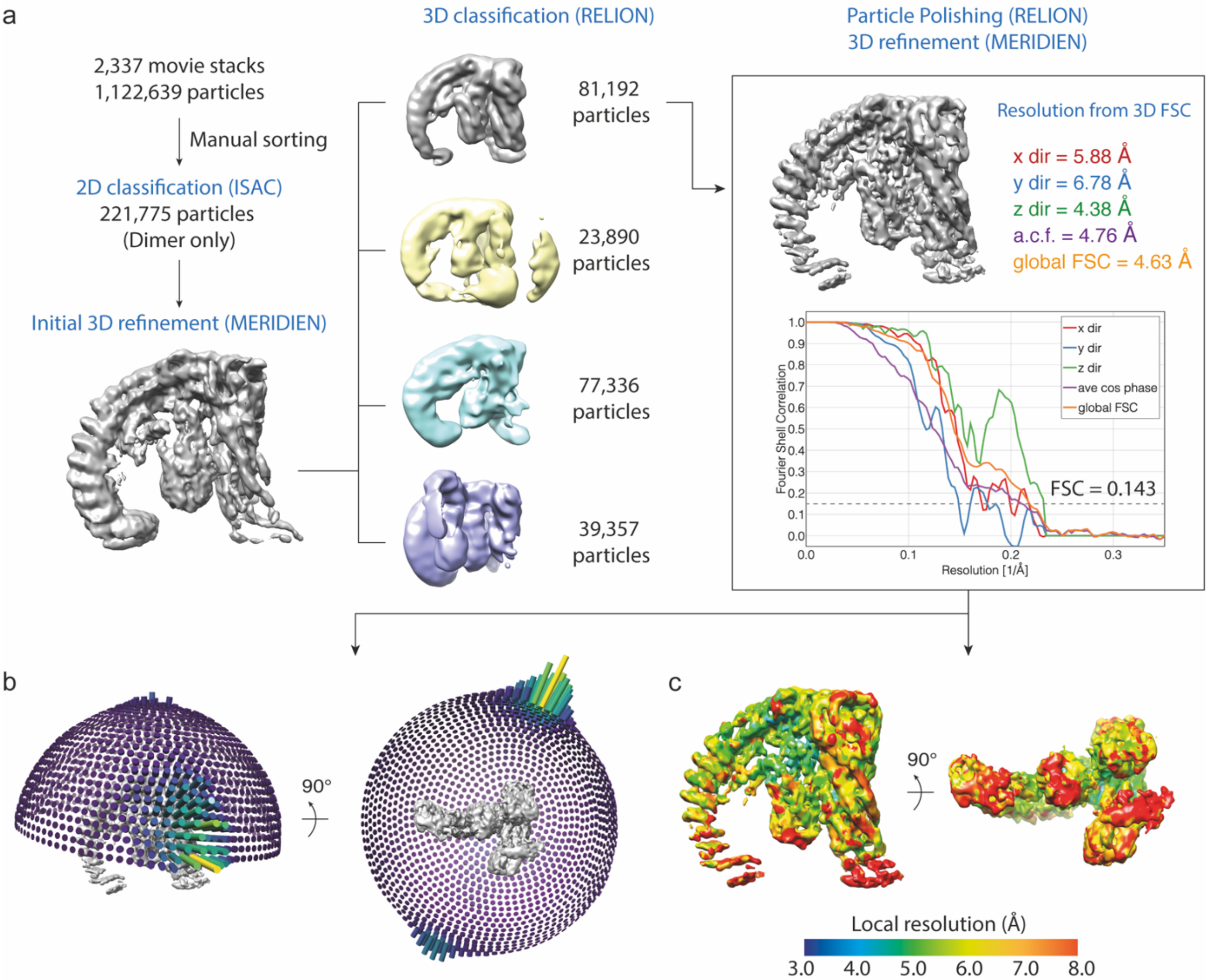

Supplementary Figure 9. CryoEM processing of δ-LIT dimer subset. a, Flowchart of data processing steps. See 'data processing' section in Methods for further details. **b**, Angular distribution for the final reconstruction of the δ-LIT dimer **c**, CryoEM density map of δ-LIT dimer colored by local resolution.

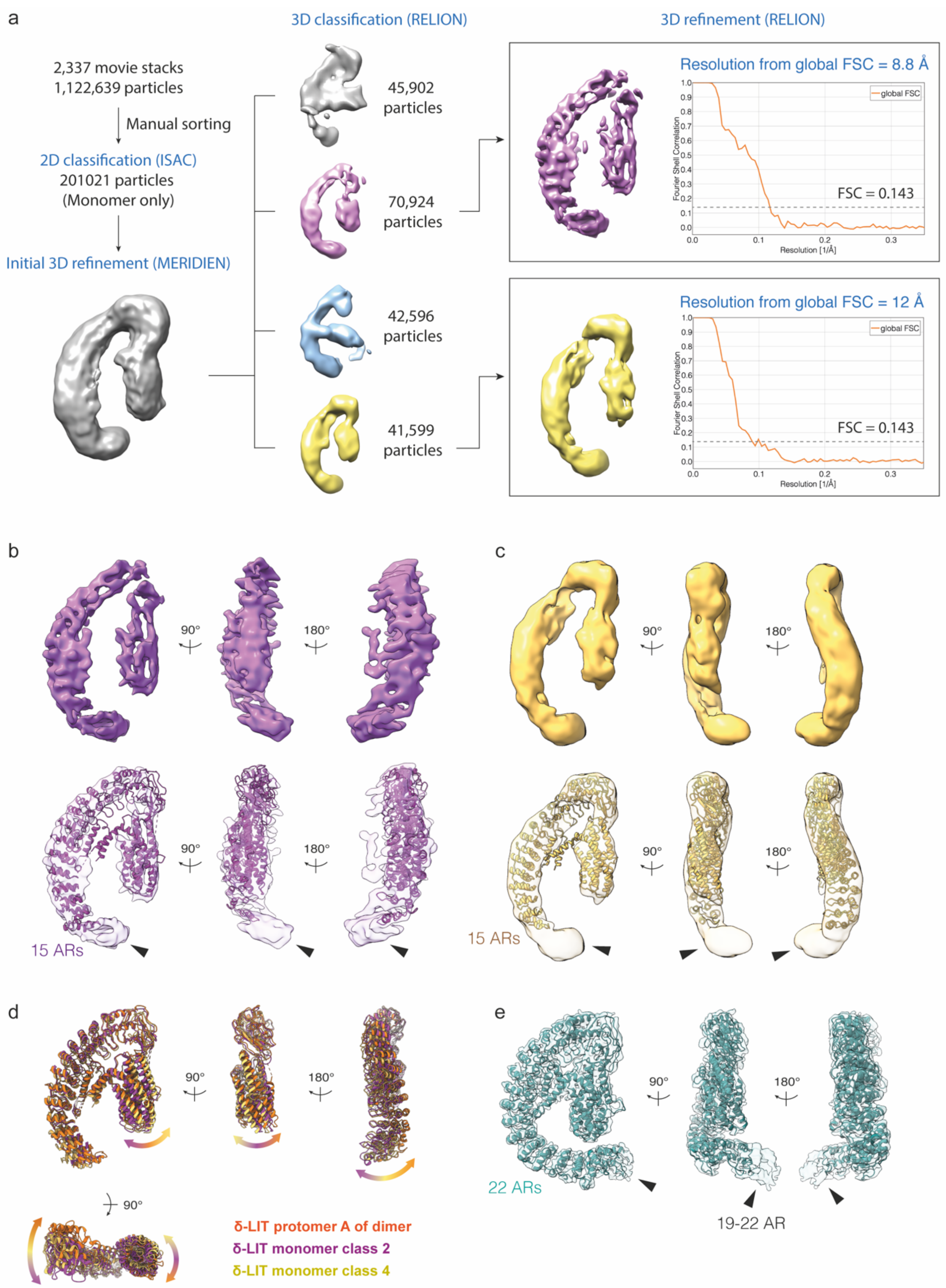

a
3D classification (RELION)
3D refinement (RELION)
2,337 movie stacks
1,122,639 particles
Manual sorting
2D classification (ISAC)
201021 particles
(Monomer only)
Initial 3D refinement (MERIDIEN)
45,902 particles
70,924 particles
42,596 particles
41,599 particles
Resolution from global FSC = 8.8 Å
FSC = 0.143
Resolution from global FSC = 12 Å
FSC = 0.143
global FSC
Fourier Shell Correlation
Resolution [1/Å]
b
90°
180°
15 ARs
c
15 ARs
d
90°
180°
δ-LIT protomer A of dimer
δ-LIT monomer class 2
δ-LIT monomer class 4
e
22 ARs
19-22 AR

Supplementary Figure 10. CryoEM processing and structural analysis of δ-LIT monomer. a, Flowchart of data processing steps. See 'data processing' section in Methods for further details. **b-c**, Final maps (upper row) upon refinement of particles of classes 2 (purple) and 4 (orange). Rigid body fitting of the molecular model of δ-LIT (obtained by the cryoEM reconstruction of the dimer) into to the low-resolution monomer maps (lower row). The EM maps showed are contoured at 6σ. Additional densities corresponding to the C-terminal inhibitory domain are indicated by black arrows. **d**, Superposition of the molecular model of the protomer A (orange) of δ-LIT dimer with the models of the individual monomers. **e**, For direct comparison, the EM map and model of mature truncated α-LCT are shown. In this case, the black arrows indicate the terminal ARs 19-22.

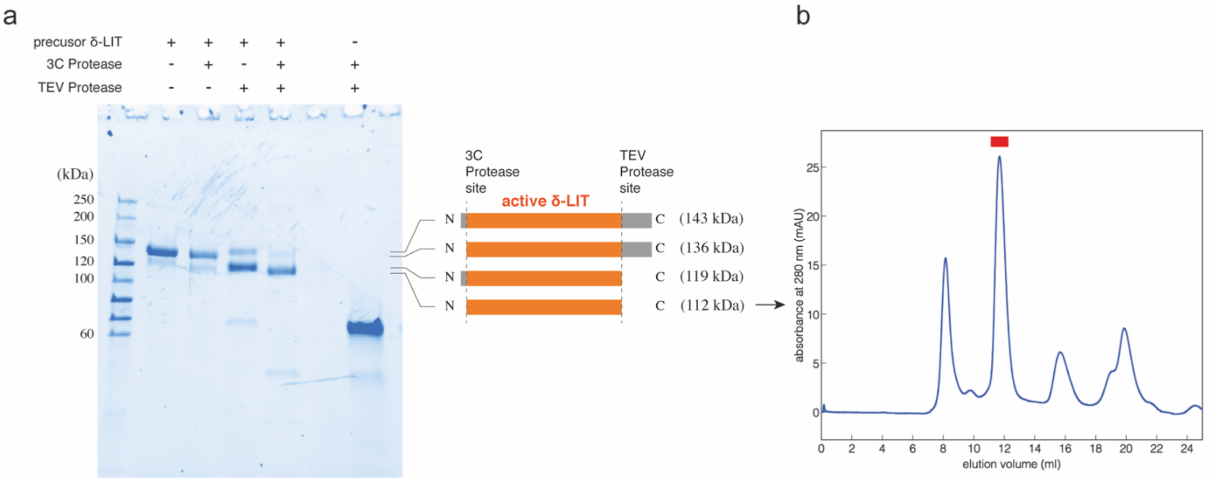

Supplementary Figure 11. **Sample preparation of mature, truncated δ-LIT. a**, SDS-PAGE of the δ-LIT sample after activation by proteolytic cleavage (lane 5) with negative controls. A total of n=3 equivalent experiment have been conducted. The expected primary structures and molecular weights are indicated. **b**, SEC profile of the sample after proteolytic cleavage. The third peak (red bar) was pooled and used for the following electrophysiologic analysis.

Current traces of precursor δ-LIT 150/150 mM KCl and 10/1 mM $CaCl_2$

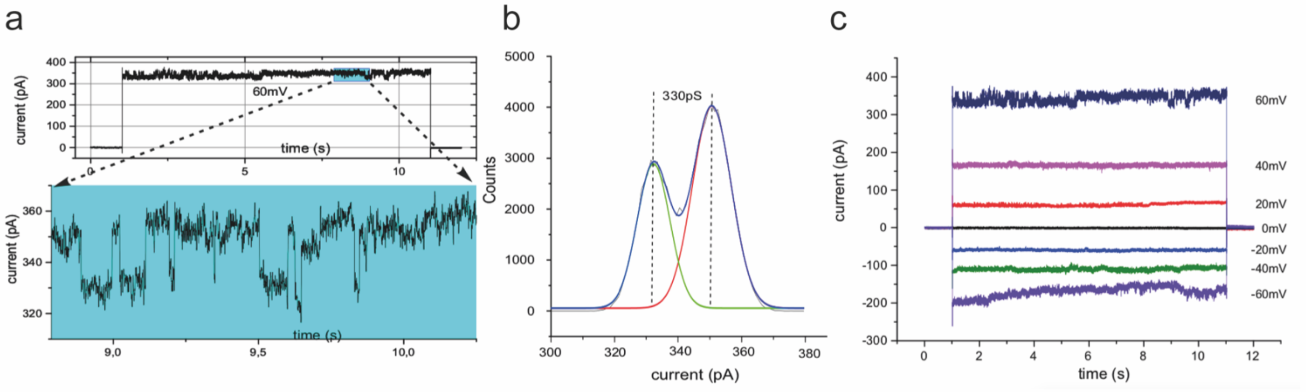

Current traces of mature δ-LIT 250/25 mM KCl and 5/0 mM $CaCl_2$

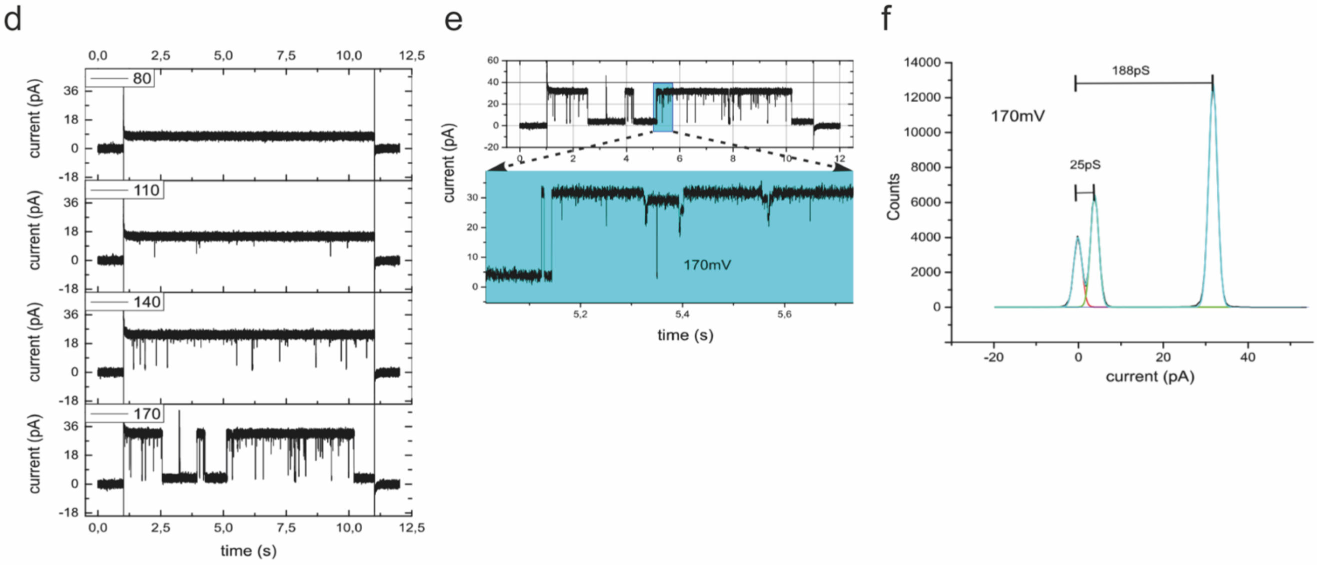

Electrophysiological characterization of mature α-LCT 150/150 mM KCl and 10/1 mM KCl

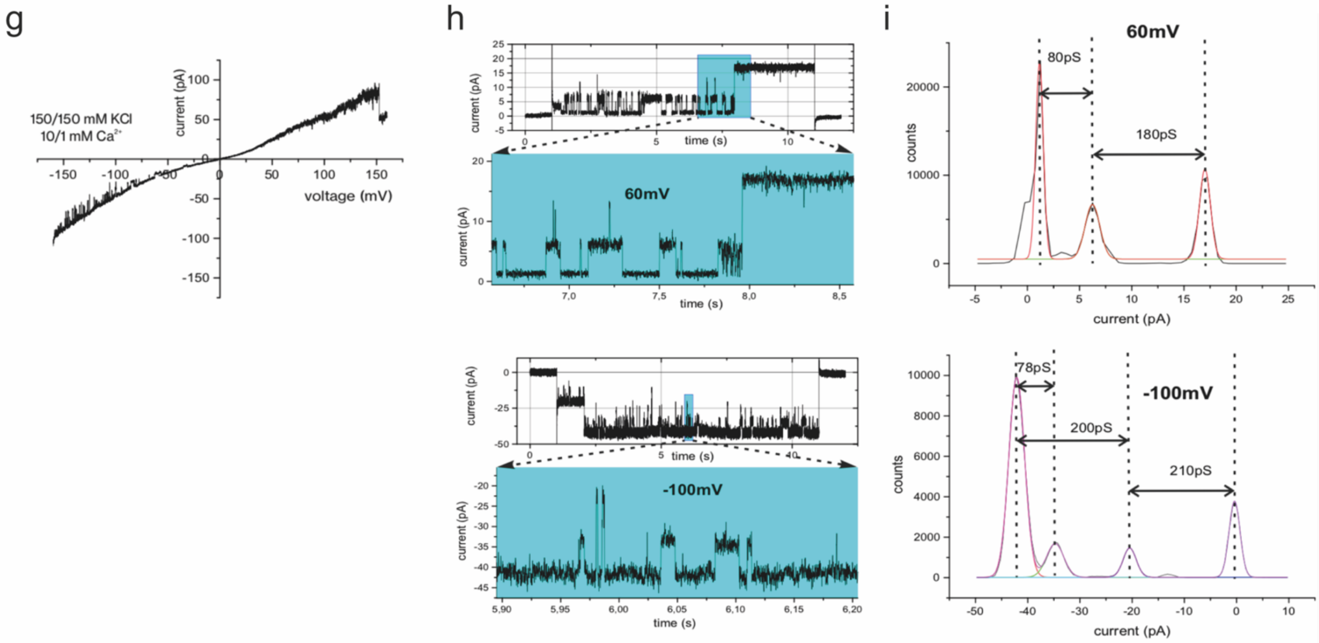

Supplementary Figure 12. **Electrophysiological analysis of latrotoxins. a,** Current recording and expansion plot from a bilayer containing the precursor δ-LIT in symmetrical 150/150 mM KCl (cis/trans) and 10/1 mM $CaCl_2$ buffer (cis/trans) in response to a voltage gate with V_{cmd}=60mV. **b,** All point current amplitude histogram from the record in (**a**). **c**, Current recordings from a bilayer containing the precursor δ-LIT in symmetrical 150/150 mM KCl and 10/1 mM $CaCl_2$ buffer (cis/trans) in response to voltage gates with the indicated amplitudes. A total of n=12 recordings similar to (c) from 3 different precursor δ-LIT preparations have been conducted. Remarkably precursor δ-LIT channel gating was rather unstructured and noisy with fast current transitions which could not be clearly resolved in amplitude or time (**a,c**). Using a simplified cylindrical model this $\bar{G}_{main}$ would correspond to a pore restriction diameter of about 1.5 nm[1,2] (c) **d**, Current recording from a bilayer containing the reconstituted mature δ-LIT in asymmetric 250/25 mM KCl and 5/0 mM $CaCl_2$ (cis/trans) buffer at the indicated V_{cmd}. A total of n=15 recordings similar to (d) at positive and negative V_{cmd} from 3 different δ-LIT preparations have been conducted. In comparison to precursor δ-LIT at all applied V_{cmd} significantly smaller overall currents were observed with the reconstituted δ-LIT pointing to a lower membrane incorporation efficiency of the solubilized mature δ-LIT protein. In addition, the current traces displayed at the given time-resolution clearly defined low noise gating patterns (**d,e**) **e**, Extension plot of the recording at V_{cmd}=170 mV. **f**, All point current amplitude histogram at V_{cmd}=170 mV of the respective recording in **d. g,** Current-voltage ramp recording from a bilayer containing the mature α-LCT in 150/150 mM KCl and 10/1 mM $CaCl_2$ (cis/trans) buffer revealing a slightly rectifying shape. A total of n=9 recordings similar to (g) from 3 different α-LCT preparations have been conducted. **h**, Current recording and extension plot in response to voltage gates of V_{cmd}= 60 mV and V_{cmd}=-100 mV. A total of n=9 recordings similar to (h) at positive and negative V_{cmd} from 3 different α-LCT preparations have been conducted. **i,** Corresponding all point current amplitude histogram at V_{cmd}= 60mV and -100mV revealing two open channel conductance states.

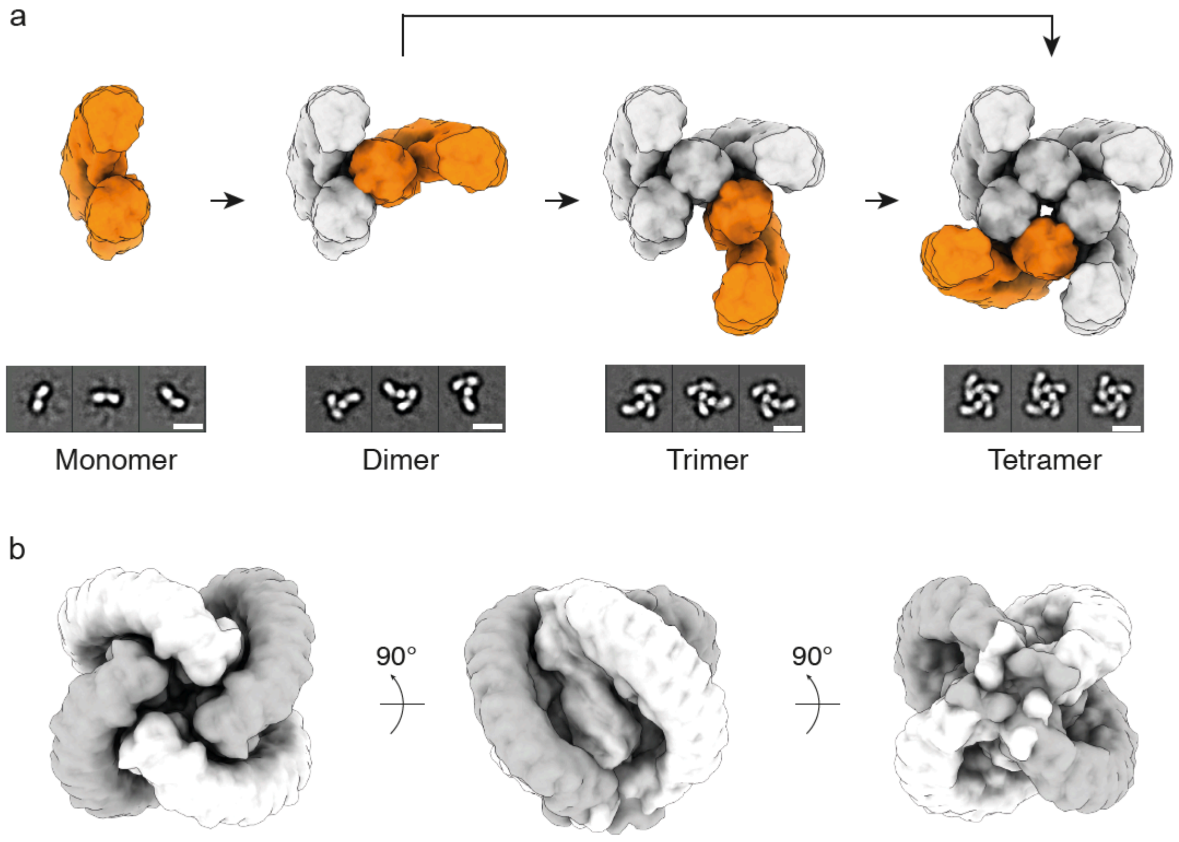

Supplementary Figure 13. a, Proposed sequential circular mechanism of tetramer formation of latrotoxin vie 1/2/3 mers. Representative 2D classes of each oligomer state from the present δ-LIT dataset are shown below the respective densities of simulated volumes filtered to 10 Å. **b**, Simulated volume of a tetramer assembled from "compact" state α-LCT monomers to demonstrate the mismatch to the 2D class averages of δ-LIT tetramers (see **a**) and the clashes between the AR domains. According to our model, conformational changes from the "compact" to "extended" state take place already during dimer formation and not after tetramer formation.

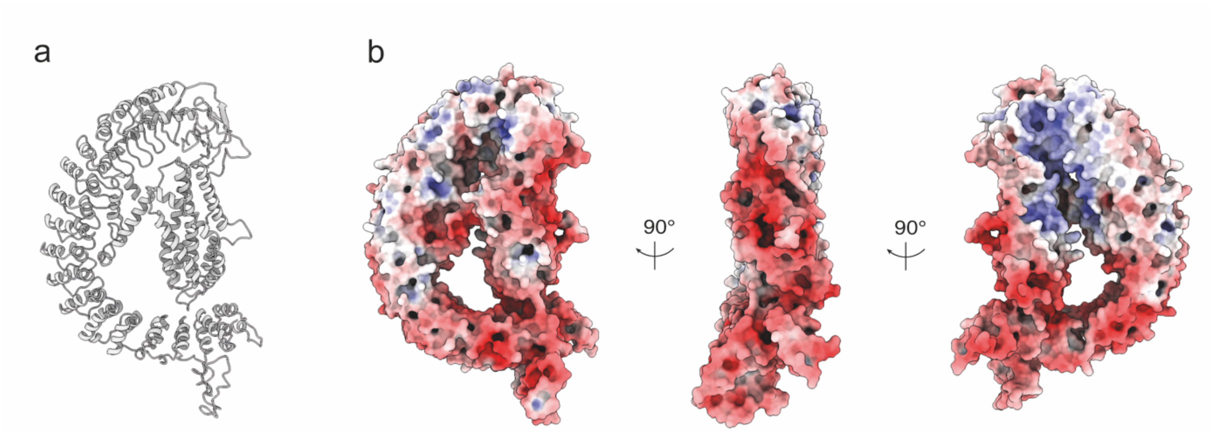

Supplementary Figure 14. Surface electrostatics of α-LTX a, Homology model of α-LTX based on the α-LCT monomer structure generated by SWISS-MODEL, using α-LCT as reference . **b**, Surface electrostatics of the α-LTX homology model at pH 7.2 calculated in APBS. Red: -10 kT/e; Blue: 10 kT/e.

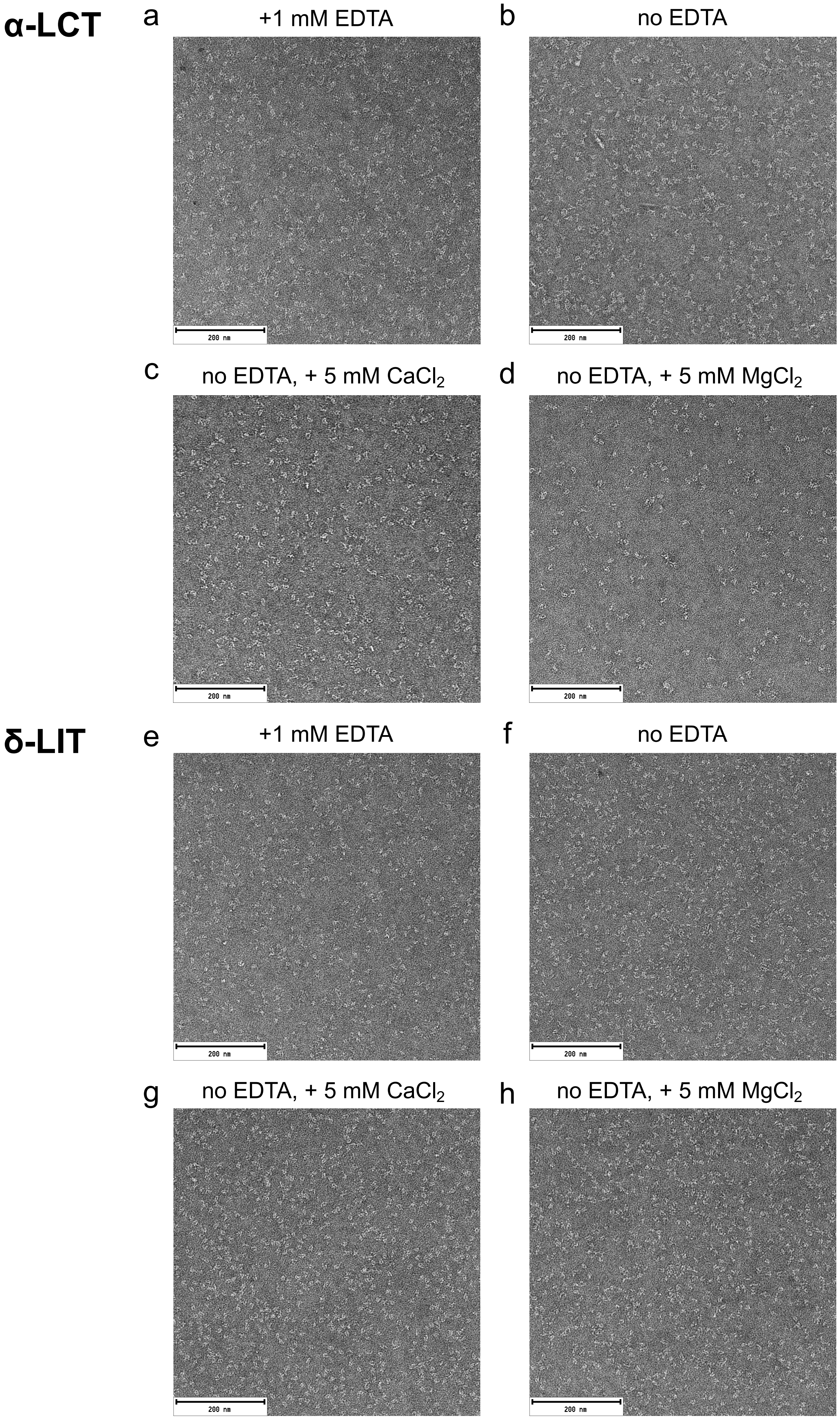
α-LCT
a
+1 mM EDTA
b
no EDTA
c
no EDTA, + 5 mM CaCl2
d
no EDTA, + 5 mM MgCl2
δ-LIT
e
+1 mM EDTA
f
no EDTA
g
no EDTA, + 5 mM CaCl2
h
no EDTA, + 5 mM MgCl2
200 nm

Supplementary Figure 15. The effect of divalent cations on LTXs a, A representative negative stain micrograph of mature α-LCT in the presence of 1 mM EDTA. **b,** α-LCT in the absence of EDTA. **c,** α-LCT in the absence of EDTA with 5 mM $CaCl_2$. **d,** α-LCT in the absence of EDTA with 5 mM $MgCl_2$. **e,** mature δ-LIT in the presence of 1 mM EDTA. **f,** δ-LIT in the absence of EDTA. **g,** δ-LIT in the absence of EDTA with 5 mM $CaCl_2$. **h,** δ-LIT in the absence of EDTA with 5 mM $MgCl_2$. A total of n=10 equivalent images of each condition have been collected. All the protein samples were adjusted to approximately 0.015 mg/ml and incubated with according additives in the presence of 100 mM Tris-HCl pH 8.0 and 150 mM NaCl at R.T. for 30 min. The negative stain EM images were acquired with a 120 kV FEI Tecnai Spirit TEX (Thermo Fisher Scientific) at a magnification of 67,000 x. Scale bar: 200 nm

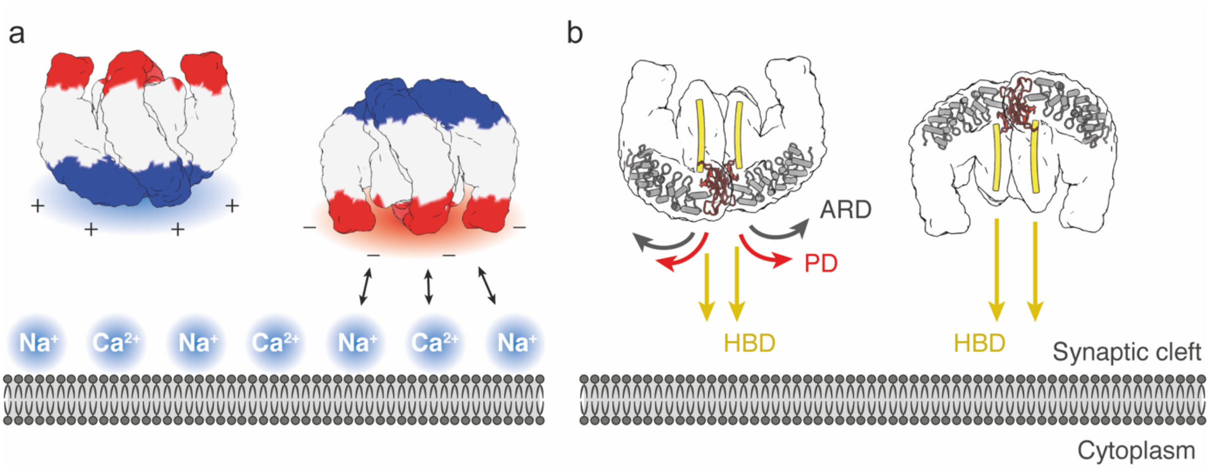

Supplementary Figure 16. Possible orientation of soluble LaTX tetramers towards the presynaptic membrane prior pore formation. Two alternative scenarios are shown side-by-side in a and b. a, The cartoon scheme of the toxin is colored according to its bipolar charge distribution. The toxin is orientated with the "claw"-tips (AR-tails) (right-image) or "claw"-head (oligimerization interface) facing the presynaptic membrane. **b,** Ribbon drawings of the predicted putative transmembrane helices of the helical bunble domain (HBD) (yellow), and the domains involved in the oligomerization interface (plug domain (PD, red) and AR1-6 of the AR-domain (ARD, Gray) are shown. Arrows indicate possible re-arrangements of domains required to allow interactions of the HBD with the membrane.

Protomer A

Domain	helix	residue
HBD	H9	Asp310
		Ser304
		Asp300
		ILE297

PD		Lys435
		Lys434
		Pro433
		Val431
		Arg430
		Lys428
		Ser371
		Glu372
		Val373
		Asn374
		Phe375
		Pro376
		Asn377
		Glun381
		Val419
		Gln420
		Gly421

Protomer B

residue	helix	Domain
Lys184	H6	HBD
Arg188		
Leu192		
Lys200	H7a	
Ala199		

Val74	H2	CD
Glu649	AR6	ARD
Asn648		
ILE647		
Ser615	AR5	
Met614		
Phe583	AR4	
Ser581		
Arg580		
Glu546	AR3	
Ala547		
Gln549	AR3	
Arg512	AR2	
Tyr511		
ILE476	AR1	
Asn477		
Lys478		
Lys480		

Supplementary Table 1. Residues which are considered to contribute to dimerization. The spatially adjacent residues are indicated with the same color in the table. The helices and domains where the residues are located are also indicated.

Oligo name	Sequence
δLIT-delDomianI-forward	gccatgggacgaggaagatggtgaaatgacc
δLIT- delDomianI-reverse	tcttcctcgtcccatggcgggccctgaaa
δLIT-TEVsite-forward	gaaaacctgtattttcagggcagcatcgttaaagaaaccaacagccgttatctgcc
δLIT-TEVsite-reverse	gctgccctgaaaatacaggttttcctccggcagggtacgacgaaac

Supplementary Table 2. Primer sequences used to producing the mature δ-LIT.

	α-LCT monomer	δ-LIT dimer
**Data collection and processing**		
Microscope	Titan Krios (X-FEG, Cs 2.7 mm)	Titan Krios (X-FEG, Cs -corrected)
Energy filter /Slit width (eV)	GIF Quantum / 20	GIF Quantum / 20
Voltage (kV)	300	300
Defocus range (um)	-1.3 to -2.5	-1.3 to -2.4
Camera	K3 super-res.	K3 super-res.
Pixcel size (Å)[a]	0.45 / 0.9	0.45 / 0.9
Total electron dose (e/Å^2)	69	58
Exposure time (sec)	3	3
Frames per movie	60	60
Nr. of images	10,439	2,339
Initial Nr. of particles	2,735,530	1,122,639
Final Nr. of particles	376,474	81,192
Map resolution (Å)[b]	4.03	4.63
B-factor for sharpening	216.70	75.01

[a] Pixel size after 2x binning used for processing

[b] Global resolution caulculated at the 'gold standard' 0.143 criterion

	α-LCT monomer	δ-LIT dimer
**Atomic model**		
Non-hydrogen atoms	7655	13435
Protein residues	1000	1715
Ligands	N/A	N/A
r.m.s.d. Bond length	0.003	0.003
r.m.s.d. Bond angles	0.756	0.681
All-atoms clash score	19.10	24.08
Rotamer outliers (%)	0	0
Ramachandran outliers (%)	0	0
Ramachandran allowed (%)	10.56	10.89
Ramachandran favored (%)	89.44	89.11
MolProbity score	2.34	2.45

Supplementary Table 3. CryoEM data collection and refinement statistics.

Supplementary References

1 Hille, B. *Ionic Channels of Excitable Membranes*. Vol. 3 (Sinauer Ass. Inc., 2001).

2 Smart, O. S., Breed, J., Smith, G. R. & Sansom, M. S. P. A novel method for structure-based prediction of ion channel conductance properties. *Biophysical Journal* **72**, 1109-1126 (1997).
