# Supplementary material for: Molecular architecture of black widow spider neurotoxins
Source: Nat Commun. 2021 Nov 29;12:6956. doi: 10.1038/s41467-021-26562-8 (PMC8630228; doi:10.1038/s41467-021-26562-8)
Supplement: Supplementary file 2 — Description of Additional Supplementary Files [file 41467_2021_26562_MOESM2_ESM.pdf]

Description of Additional Supplementary Files

Supplementary Movie 1. The cryoEM map and the overall structure of α-LCT monomer.

Supplementary Movie 2. The cryoEM map and the overall structure of δ-LIT dimer.

Supplementary Movie 3. Putative conformational change during oligomerization. The four conformations, i.e., the “compact” (sea green; α-LCT), an intermediate (cyan: α-LCT) and the “extended” state (orange: δ-LIT protomer A; gray: δ-LIT protomer B) are shown sequentially. The morphing occurs between the “compact” conformation of the α-LCT monomer and the “extended” conformation of δ-LIT protomer B. The conformational change of the overall structure is shown followed by close-up views of the helical bundle-, connector- and AR-domain.
