# Supplementary material for: Molecular architecture of black widow spider neurotoxins
Source: Nat Commun. 2021 Nov 29;12:6956. doi: 10.1038/s41467-021-26562-8 (PMC8630228; doi:10.1038/s41467-021-26562-8)
Supplement: Supplementary file 6 — Reporting summary [file 41467_2021_26562_MOESM6_ESM.pdf]

nature research

Corresponding author(s): Christos Gatsogiannis

Last updated by author(s): 23.09.2021

Reporting Summary

Nature Research wishes to improve the reproducibility of the work that we publish. This form provides structure for consistency and transparency in reporting. For further information on Nature Research policies, see our Editorial Policies and the Editorial Policy Checklist.

Statistics

For all statistical analyses, confirm that the following items are present in the figure legend, table legend, main text, or Methods section.

n/a	Confirmed	
☐	☒	The exact sample size (n) for each experimental group/condition, given as a discrete number and unit of measurement
☐	☒	A statement on whether measurements were taken from distinct samples or whether the same sample was measured repeatedly
☒	☐	The statistical test(s) used AND whether they are one- or two-sided<br>*Only common tests should be described solely by name; describe more complex techniques in the Methods section.*
☒	☐	A description of all covariates tested
☒	☐	A description of any assumptions or corrections, such as tests of normality and adjustment for multiple comparisons
☒	☐	A full description of the statistical parameters including central tendency (e.g. means) or other basic estimates (e.g. regression coefficient) AND variation (e.g. standard deviation) or associated estimates of uncertainty (e.g. confidence intervals)
☒	☐	For null hypothesis testing, the test statistic (e.g. F, t, r) with confidence intervals, effect sizes, degrees of freedom and P value noted<br>*Give P values as exact values whenever suitable.*
☒	☐	For Bayesian analysis, information on the choice of priors and Markov chain Monte Carlo settings
☒	☐	For hierarchical and complex designs, identification of the appropriate level for tests and full reporting of outcomes
☒	☐	Estimates of effect sizes (e.g. Cohen's d, Pearson's r), indicating how they were calculated

Our web collection on statistics for biologists contains articles on many of the points above.

Software and code

Policy information about availability of computer code

Data collection	EPU ver.2.8
Data analysis	TranSPHIRE ver.10 July 2020, SPHIRE ver 1.3 (ISAC, RVIPER, MERIDIEN are implemented in the SPHIRE package and do not have individual version numbers), MotionCor2 ver.1.3.0, CTFFIND4 ver.4.1.10, crYOLO ver. 1.4, RELION ver 3.1, PHENIX ver.1.19.2, COOT ver.1.0, Pymol ver. 2.2.2, UCSF ChimeraX v01.er.1.0 and 1.1.1, ISOLDE ver.1.1.0, SWISS-MODEL ver.04 Jan. 2021, CCP4 ver 1.0, 3DFSC server ver.3.0, RaptorX Property ver.4.0, Clustal Omega ver.1.2.2, TMHMM server ver.2.0, APBS ver3.0.0, ESPript ver.3.0, Adobe Illustrator ver.25.2.0, Heka-PatchMaster2x73.2, Origin package OriginPro ver.8.5.1, MATLAB Mathcad ver.15.0.

For manuscripts utilizing custom algorithms or software that are central to the research but not yet described in published literature, software must be made available to editors and reviewers. We strongly encourage code deposition in a community repository (e.g. GitHub). See the Nature Research guidelines for submitting code & software for further information.

Data

Policy information about availability of data

All manuscripts must include a data availability statement. This statement should provide the following information, where applicable:

- Accession codes, unique identifiers, or web links for publicly available datasets
- A list of figures that have associated raw data
- A description of any restrictions on data availability

The amino acid sequences used for alignment in this study are available in the Universal Protein Resource, UniProt (UniprotKB IDs: Q9XZC0, Q25338, Q02989, L7XCU0, P23631, L7XDS4, G0LXV8, P0DJE3, L7X8P2). The cryoEM datasets of α-LCT and δ-LIT generated in this study have been deposited in EMPIAR under accession codes EMPIAR-10827 and EMPIAR-10828. The cryoEM maps of α-LCT monomer and δ-LIT dimer have been deposited in the Electron Microscopy Data Bank (EMDB) under accession codes EMD-13642 and EMD-13643. The coordinates of the corresponding models have been deposited to the Protein Data Bank

(PDB) under accession codes 7PTX and 7PTY. The full sequences of the plasmids used in this study and the raw electrophysiological data as tab-separated asci-files of the respective individual figures are provided in the Source Data. Other data are available from the corresponding author upon reasonable request.

Field-specific reporting

Please select the one below that is the best fit for your research. If you are not sure, read the appropriate sections before making your selection.

- [x] Life sciences
- [ ] Behavioural & social sciences
- [ ] Ecological, evolutionary & environmental sciences

For a reference copy of the document with all sections, see nature.com/documents/nr-reporting-summary-flat.pdf

Life sciences study design

All studies must disclose on these points even when the disclosure is negative.

Sample size	Protein concentration was measured by the Bradford method (Bradford protein assay kit, Bio-Rad). For EM experiments, the sample size was experimentally determined based on particles distribution on micrographs. Examples are shown in supplementary figures 2b and 8d. The electrophysiology experiments were performed multiple times with similar results and further inclusion of data did not change the results.
Data exclusions	No data were excluded
Replication	For EM experiments, several cryoEM grids were prepared. We selected the highest quality grid for dataset collection. Electrophysiology experiments were repeated multiple times on different days. All replication attempts were successful.
Randomization	Randomization is not relevant to this structural study and the electrophysiology experiments.
Blinding	Blinding is not relevant because there are no animals or patients involved in this structural study.

Reporting for specific materials, systems and methods

We require information from authors about some types of materials, experimental systems and methods used in many studies. Here, indicate whether each material, system or method listed is relevant to your study. If you are not sure if a list item applies to your research, read the appropriate section before selecting a response.

Materials & experimental systems

n/a	Involved in the study	
x		Antibodies
	x	Eukaryotic cell lines
x		Palaeontology and archaeology
x		Animals and other organisms
x		Human research participants
x		Clinical data
x		Dual use research of concern

Methods

n/a	Involved in the study	
x		ChIP-seq
x		Flow cytometry
x		MRI-based neuroimaging

Eukaryotic cell lines

Policy information about cell lines

Cell line source(s)	Sf9 cells: purchased from Oxford Expression Technologies (Cat. No. 600100)<br>Hi5 cells: purchased from ThermoFisher Scientific (Cat. No B85502)
Authentication	The cell lines were not authenticated.
Mycoplasma contamination	Cell lines were not tested for mycoplasma contamination.
Commonly misidentified lines (See ICLAC register)	No commonly misidentified cell lines were used in the study.
