# Supplementary figures and images for: Molecular architecture of black widow spider neurotoxins
Source: Nat Commun. 2021 Nov 29;12:6956. doi: 10.1038/s41467-021-26562-8 (PMC8630228; doi:10.1038/s41467-021-26562-8)

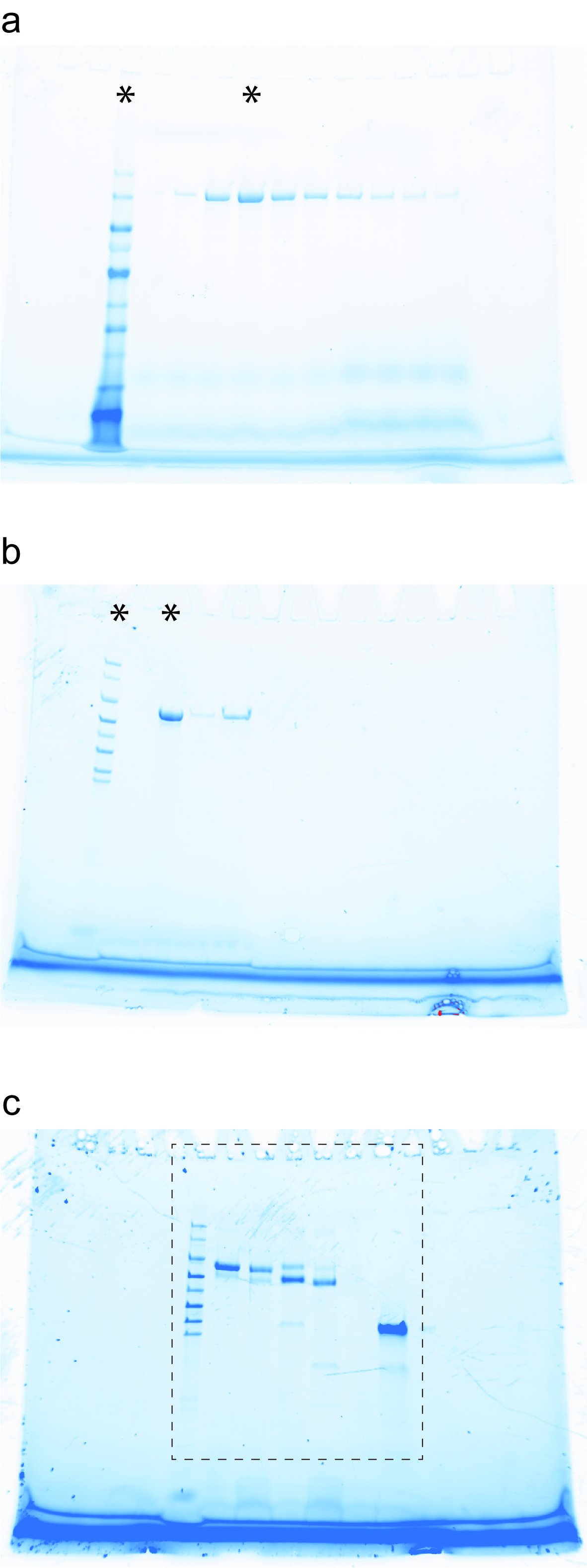

Supplement: Supplementary file 7 — Source Data [file 41467_2021_26562_MOESM7_ESM.zip › Extended_data_5.tif]
